# Erythroid-transdifferentiated myeloid cells promote portal vein tumor thrombus in hepatocellular carcinoma

**DOI:** 10.7150/thno.82907

**Published:** 2023-07-31

**Authors:** Wei-Hang Zhu, Jie Chen, Run-Kai Huang, Yuan Zhang, Ze-Xuan Huang, Xiu-Qing Pang, Bo Hu, Yang Yang, Xing Li

**Affiliations:** 1 Department of Medical Oncology, the Third Affiliated Hospital of Sun Yat-sen University, 600 Tianhe Road, Guangzhou 510630, China.; 2 Guangdong Key laboratory of Liver Disease Research, the Third Affiliated Hospital of Sun Yat-sen University, 600 Tianhe Road, Guangzhou 510630, China.; 3 Department of Obstetrics, the Third Affiliated Hospital of Sun Yat-sen University, 600 Tianhe Road, Guangzhou 510630, China.; 4 Department of Infectious Diseases, the Third Affiliated Hospital of Sun Yat-sen University, 600 Tianhe Road, Guangzhou 510630, China.; 5 Department of Laboratory Medicine, the Third Affiliated Hospital of Sun Yat-sen University, 600 Tianhe Road, Guangzhou, 510630, China.; 6 Department of Hepatic Surgery and Liver Transplantation Center & Guangdong Provincial Key Laboratory of Liver Disease Research, the Third Affiliated Hospital, Sun Yat-sen University, 600 Tianhe Road, Guangzhou, 510630, China.

**Keywords:** Portal vein tumor thrombus, Coagulation, CD45^+^ EPC, Hepatocellular carcinoma, Vascular endothelial cells

## Abstract

**Rationale**: Hepatocellular carcinoma (HCC) is primarily characterized by a high incidence of vascular invasion. However, the specific mechanism underlying portal vein tumor thrombus (PVTT) in HCC remains unclear. As a consequence of myeloid cell developmental arrest, CD71^+^ erythroid progenitor cells (EPCs) and myeloid-derived suppressor cells play important roles in HCC; however, their roles in PVTT remain unclear.

**Methods:** The role of CD71^+^ EPCs in the HCC tumor microenvironment (TME) was evaluated via morphological, RNA-sequencing, enzyme-linked immunosorbent assay, and flow cytometric analyses. Co-culture techniques were employed to assess the CD45^+^ EPCs and their vascular compromising effect. Additionally, the PVTT-promoting function of CD45^+^ EPCs was explored *in vivo* in a murine model.

**Results:** The CD45^+^EPCs in HCC tissues exhibited increased myeloid cell features, including morphology, surface markers, transforming growth factor (TGF)-β generation, and gene expression, compared with those in circulation. Hence, a large proportion of CD45^+^EPCs, particularly those in TMEs, comprise erythroid-transdifferentiated myeloid cells (EDMCs). Additionally, the expression of C-C chemokine receptor type 2 (CCR2) mRNA was upregulated in CD45^+^EPCs within the TME. Tumor macrophages from HCC tissues induced substantial migration of CD45^+^EPCs in a dose-dependent manner. Meanwhile, results from immunofluorescence analyses revealed that these two cell types are positively associated in the TME and circulation. That is, EDMCs are chemoattracted by HCC macrophages mainly via CCR2 from CD45^+^ EPCs in the circulation. Additionally, the expressions of FX, FVII, FGB, C4b, CFB, and CFH were elevated in CD45^+^EPCs within the TME compared with those in the spleen. The CD45^+^EPCs from the HCC TME promoted vessel endothelial cell migration and compromised tube formation through TGF-β and FGB, respectively. Additionally, CD45^+^EPCs from the TME induced HCC cell migration. HCC macrophage-induced CD45^+^EPCs to exhibit higher levels of FX, FVII, FGB, and TGF-β. Meanwhile, upregulation of CCAAT/enhancer binding protein beta expression induced FGB and TGF-β generation in CD45^+^EPCs in the TME. WTAP, a major RNA m^6^A writer, stabilized *FX* and *FVII* mRNA and enhanced their nuclear export in CD45^+^EPCs from the TME. CD45^+^EPCs from the TME were positively associated with PVTT and poor prognosis. Splenectomy reduced the level of CD45^+^EPCs in the circulation and TME, as well as the incidence of microvascular invasion. The incidence of microvascular invasion increased following the transfer of HCC tissue CD45^+^EPCs to splenectomized HCC-bearing mice.

**Conclusions:** The CD45^+^EPCs enriched in the HCC microenvironment are EDMCs, which are induced by HCC macrophages to migrate from the circulation to the TME. Subsequently, EDMCs promote PVTT by compromising the blood vessel endothelium, aggravating coagulation, and promoting HCC cell migration.

## Introduction

A high incidence of vascular invasion and low probability of extrahepatic metastasis are the primary characteristics of hepatocellular carcinoma (HCC) 1. Portal vein tumor thrombus (PVTT) is found in 16-30% of patients with HCC at the time of diagnosis 1. The destruction of the blood vessel endothelium and activation of the coagulation process are the key mechanisms underlying PVTT formation. Tumors secrete a variety of procoagulant substances (including tissue factors, planopodia proteins, and plasminogen activator inhibitors) and macrovesicles carrying phosphatidylserine to initiate coagulation and activate platelets in various ways (ADP and thrombin), resulting in the formation of thrombus 2-4. Tumors can also directly invade or diffuse into blood vessels, activating platelets and promoting neutrophils to produce extracellular trapping nets, which in turn activate vessel endothelial cells (VECs) to promote coagulation 3, 5. However, the causes responsible for the high incidence of PVTT in HCC remain unclear 6.

Myeloid cell differentiation and developmental arrest are characteristics of immune dysregulation in patients with cancer 7, 8. Regarding erythroid hematopoiesis, extramedullary hematopoiesis occurs during cancer and anemia 7, 9, 10. CD71^+^ erythroid progenitor cells (EPCs) are produced during the differentiation of megakaryocyte-erythrocyte progenitor cells into erythrocytes 11, 12, which are a new type of immune cell that can promote the re-occurrence and development of tumors 7, 13. The number of EPCs in HCC tissue is positively correlated with PVTT 13. In myeloid hematopoiesis, the maturation of granulocyte-monocyte progenitor cells is blocked, resulting in the accumulation of myeloid-derived suppressor cells (MDSCs) 8, 14. MDSCs are also associated with prognosis in HCC and are reported to induce vascular VEC migration 15. VEC migration may destroy the endodermis and promote PVTT 16. Recently, Long et al. found that tumors induced a subset of CD45^+^ EPCs to differentiate into a cellular “erythroid-myeloid hybrid” population, named erythroid-differentiated myeloid cells (EDMCs) 17, which may present a connection between MDSCs and EPCs. Thus, EDMCs may play a critical role in PVTT development.

In the present study, we investigated the presence of EDMCs in the HCC microenvironment, evaluated their role in the development of PVTT, and assessed the potential underlying mechanisms and clinical applications.

## Methods

### Patients and healthy donors

Between June 2019 and September 2022, 75 patients with chronic hepatitis-related HCC were enrolled in this study. The patients underwent treatment for HCC at the Third Affiliated Hospital of Sun Yat-sen University (SYSU), Guangzhou, China. A total of 19 healthy adult donors and 6 infants were included as controls. HCC diagnosis was confirmed based on pathological findings or the American Association for the Study of Liver Disease radiological criteria using either computed tomography or magnetic imaging resonance results. All patients and healthy controls were screened for serum human immunodeficiency virus antibodies, hepatitis B surface antigen, hepatitis C virus antibodies, hepatitis D virus antigens, and hepatitis D virus antibodies. Patients and healthy controls who were positive for human immunodeficiency virus, hepatitis infection (except for hepatitis B virus), or other acute infections (including pneumonia and urinary tract infections); were pregnant; had received systemic corticosteroids, immunosuppressive agents, or anti-cancer therapies; or had a fever were excluded from this study.

### Mice

C57BL/6 mice (male, 6 weeks old) were purchased from the GuangDong Medical Laboratory Animal Center (Guangzhou, China). All mouse experiments were conducted at pathogen-free facilities.

### Cell culture

Hepa 1-6 (ATCC CRL-1830) and C166 (ATCC CRL-2581) cell lines were purchased from the American Type Culture Collection (ATCC). They were cultured in DMEM (Invitrogen, Carlsbad, CA, USA) with 10% (v/v) fetal bovine serum (Cegrogen Biotech, Germany) supplemented with 1% (v/v) antibiotics as routine. All cells were grown in a humidified incubator at 37 °C with 5% CO_2_. All cell lines were authenticated 3 months before the beginning of the study based on viability and morphology by the suppliers. Cells had not been in culture for longer than 2 months.

### Western blotting

Total protein was extracted from cells using pre-cooled RIPA buffer (Beyotime, Shanghai, China) containing protease and phosphatase inhibitors (Thermo Fisher Scientific, Waltham, MA, USA). Protein quantification was conducted with the Bicinchoninic Acid Protein Assay Kit (Thermo Fisher Scientific). An equal amount of protein samples was separated by 4-12% SDS-PAGE (GenScript, Nanjing, China) and then transferred to 0.45-μm PVDF membranes (Millipore, Billerica, MA, USA). After being blocked using 5% (v/v) non-fat milk in TBST for 1 h, membranes were incubated with corresponding primary antibodies at 4 °C overnight. Next, they were washed with TBST thrice and incubated with HRP-conjugated secondary antibodies for 1 h at room temperature. The immunoblots were detected with an imaging system (Bio-Rad, Hercules, CA, USA) using an enhanced chemiluminescence detection kit (Servicebio, Wuhan, China). GAPDH and α-tublin were selected to be the loading controls.

### m^6^A dot blot assay

Total RNA was isolated as described above. RNA samples dissolved in 3× volume of RNA incubation buffer were denatured at 65 °C within 5 min. Then, 100 ng RNA was loaded onto an Amersham Hybond-N+ membrane (GE Healthcare, Chicago, IL, USA). The membrane was UV-crosslinked for 60 min and washed with PBST. After being blocked with 5% (v/v) non-fat milk, the membrane was incubated with specific m^6^A antibody (1:1000, Millipore) overnight at 4 °C. Dot blots were hatched with horseradish peroxidase (HRP)-conjugated anti-mouse immunoglobulin G (IgG) for 1 h before visualization with an imaging system (Bio-Rad). Next, the membrane was stained with 0.2% (v/v) methylene blue (Sangon Biotech, China) and scanned to determine the total content of input RNA.

### Quantification of total m^6^A RNA

The m^6^A content of 200 ng RNA extracted from the indicated cells was analyzed using the EpiQuik m^6^A RNA Methylation Quantification Kit (Colorimetric) (Epigentek, Farmingdale, NY, USA, P-9005-48) following the manufacturer's instructions. The m^6^A level was quantified by measuring the absorbance of each well at 450 nm, and the standard curve was then used to calculate the m^6^A level.

### Real-time quantitative reverse transcription PCR (qRT-PCR)

RNA was extracted using the Multisource Total RNA Miniprep Kit (Axygen, Union City, CA, USA). The purity and concentration of RNA were determined by measuring absorbance at A260/280 nm using Nanodrop 2000 (Thermo Fisher Scientific). qRT-PCR was performed using commercially available primers (Table S1) and SYBR Premix Ex Taq II (Code, DRR081; Takara Biotechnology Co. Ltd., Dalian, China).

Fluorescence for each cycle was quantitatively analyzed using the ABI Prism 7000 sequence detection system (Life Technologies, Carlsbad, CA, USA). The results are reported as relative expression, normalized with *GADPH* as a housekeeping gene endogenous control, and expressed in arbitrary units.

### RNA decay assay

To evaluate RNA stability, an RNA decay assay was conducted. Cells were cultured in four 6-well plates followed by a treatment of *Wtap* knockdown or overexpression. Then, actinomycin D (Aladdin, Shanghai, China; A113142) was added into each well with a final concentration of 5 μg/mL, and cells were collected after 0, 3, and 6 h. Subsequentially, total RNA was isolated and subjected to qRT-PCR for quantifying the relative abundance of F7 and F10 mRNA (relative to 0 h).

### Methylated RNA immune precipitation (MeRIP)-qPCR

A MeRIP kit (PerxcyBiology, Guangzhou, China) was used for MeRIP analysis of indicated CD45^+^EPCs. Then, 5 mg target m^6^A recognition protein antibody or rat IgG with magnetic beads (Millipore) pre-coated with cell lysate (2×10^6^ cells) was incubated overnight at 4 °C. Beads containing immunoprecipitated RNA-protein complexes were treated with protease K to remove excess protein. Then, the mRNA of interest was purified with TRIzol (Thermo Fisher Scientific), and the indicated gene was detected by RT-qPCR.

### RNA sequencing data analysis

CD45^+^CD235^+^CD71^+^ cells from peripheral blood mononuclear cells and HCC tissue of one HCC patient were sorted using a FACSAria III cell sorter (BD Bioscience, Franklin Lakes, NJ, USA). CD45^+^TER119^+^CD71^+^ cells and CD45^-^TER119^+^CD71^+^ cells from bone marrow (BM), spleen, and tumor tissues from the orthotopic HCC mouse model were also sorted using the FACSAria III cell sorter (BD Bioscience). The sorting purity was > 95%. RNA sequencing was performing using the BGIseq 500 platform (BGI, Shenzhen, China). Single-end read runs were used with read lengths up to 50 bp in high output mode and total read count of 20 M. Data were aligned using RSEM v1.2.12 against the mm10 genome and gene-level read counts, and TPM values at the gene level were estimated for ensemble transcriptome assembly. Samples with at least 80% aligned reads were analyzed. DESeq2 was used to estimate significance between any two experimental groups. Overall changes were considered significant if samples had a p value of < 0.05 with an additional threshold on fold change (FC) ([log_2_FC] > 1) taken to generate the final gene set.

### Patient follow-up and statistical analysis

Patients were followed up at least every 3 months until death. Overall survival (OS) was the endpoint that was calculated from the first day of treatment to death. Progression-free survival (PFS) was calculated from the first day of therapy to the day of tumor progression, death, or the last examination. Variables in different groups were compared using the χ^2^ test (or Fisher's exact test, if indicated) and the *t*-test or nonparametric Mann-Whitney U test. OS and PFS were calculated using the Kaplan-Meier method, and differences were compared using the log-rank test. For *in vitro* experiments, statistical analyses were conducted using unpaired *t*-tests. Correlations between different parameters were analyzed using the Spearman's rank test or logistic regression. The criterion for statistical significance was set at α = 0.05, and all p-values were based on two-sided tests. Statistical tests were performed using GraphPad Prism version 9.0, R statistical software version 3.6.0, and SPSS Statistics 25.0.

Descriptions of the other methods are provided in the Supplementary Material. The antibodies and reagents used are listed in Table S2.

### Study approval

All research was conducted in accordance with the principles of the Declaration of Helsinki. This study was approved by the Clinical Ethics Review Board of the Third Affiliated Hospital of SYSU (approval no. [2021]02-119). Written informed consent was obtained from all patients, healthy donors, and parents of the infants at the time of admission. Experiments involving mice were approved by the Institutional Animal Care and Use Committee of the Third Affiliated Hospital of SYSU.

## Results

### EPCs enriched in the HCC microenvironment were EDMCs

**Figure 1:**
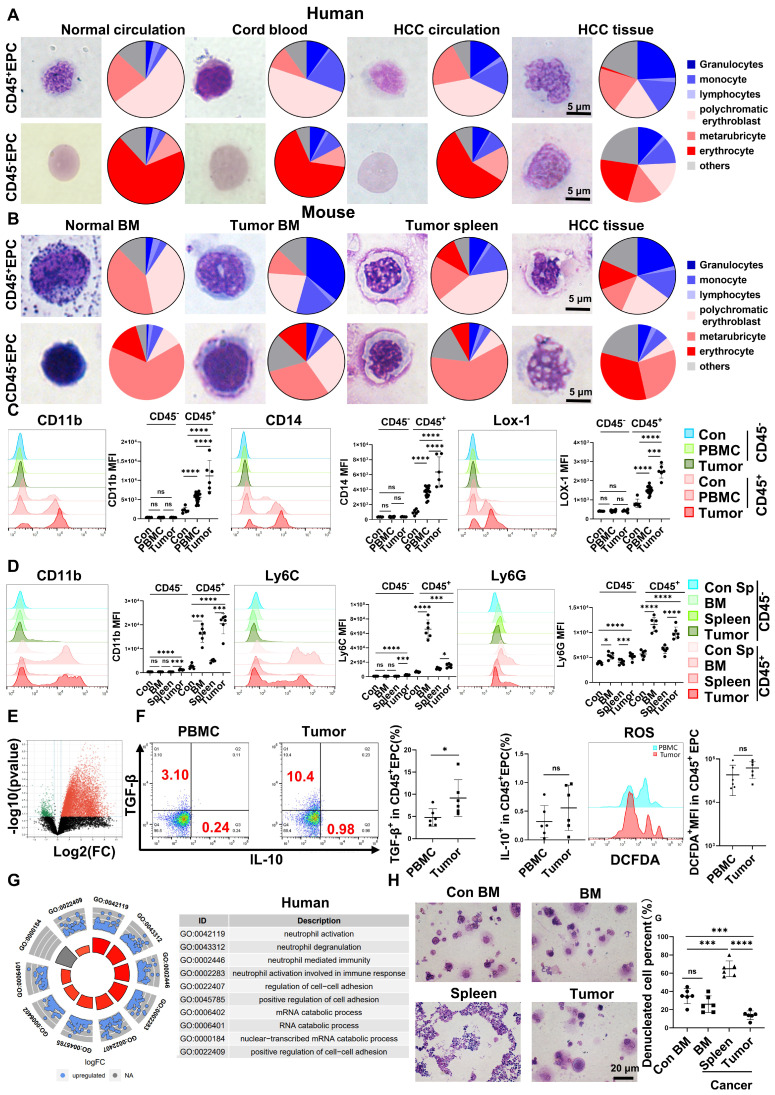
** Erythroid progenitor cells (EPCs) enriched in the hepatocellular carcinoma (HCC) microenvironment were erythroid-transdifferentiated myeloid cells (EDMCs). (A, B)** Wright-Giemsa staining of CD45^+^EPCs and CD45^-^EPCs from the circulation of healthy donor, cord blood of healthy infants, and circulation and HCC tissues of HCC patients (A), as well as those in bone marrow (BM) of tumor-free mice and BM, spleen, and HCC tissues of tumor-bearing mice (B). Cells were classified according to their morphology with the proportion of each cell type illustrated in a pie chart. **(C)** Representative and statistical analysis of cumulative fluorescence intensity (MFI) of human MDSC markers (CD11b, CD14, and LOX-1) on CD45^-^EPCs and CD45^+^EPCs from cord blood mononuclear cells (CBMCs) (n = 6), peripheral blood mononuclear cells (PBMCs) (n = 19) and tumor tissues (n = 6) of HCC patients, CBMCs were the control. **(D)** Representative and statistical analysis of MFI of mouse MDSC markers (CD11b, Ly6C, and Ly6G) on CD45^-^EPCs and CD45^+^EPCs from the spleen of tumor-free mice, and BM, spleen and HCC tissues of tumor-bearing mice. Spleens of tumor-free mice were the control. **(E)** Whole-transcriptome analysis using RNA-seq was conducted on CD45^-^EPCs and CD45^+^EPCs from three samples of HCC tissues from tumor-bearing mice. Volcano plots displaying differentially expressed genes with red dots represent the upregulated expressed transcripts (p < 0.05, [log_2_ fold change] > 1) and green dots represent the transcripts whose expression downregulated (p < 0.05, [log_2_ fold change] > 1). **(F)** Representative and statistical analysis of flow cytometer analysis of TGF-β, IL-10, and ROS (tested by DCFDA) on CD45^+^EPCs from PBMC (n = 6) and tumor tissues (n = 6) of HCC patients. **(G)** Gene ontology analysis of differentially expressed genes between CD45^+^EPCs from the circulation and tumor tissue in one patient with HCC. **(H)** Typical figure illustrated by Wright-Giemsa staining and statistical analysis of denucleated red cells induced by erythropoietin from CD45^+^EPCs from BM, spleen or tumor tissues of tumor-bearing mice and BM of tumor-free mice in erythroid cell development culture system *in vitro*. ****p < 0.0001; ***p < 0.001; **p < 0.01; *p < 0.05. con: control; DCFDA: 2′,7′-dichlorofluorescein diacetate; IL: interleukin; ns: non-significant; ROS: reactive oxygen species; TGF: transforming growth factor.

EPCs (CD71^+^CD235^+^ for human cells; CD71^+^Ter119^+^ for mouse cells) from circulation, BM, and tumor tissues were highly heterogeneous in morphology, presenting similar morphologies to those of granulocytes, monocytes, polychromatic erythroblasts, metarubricytes, erythrocytes, and atypical cells. Compared with those from tumor-free controls, EPCs from patients with cancer and HCC tumor-bearing mice presented an increased number of myeloid cell and relatively immature erythroid cell morphologies, especially in CD45^+^ EPCs. The CD45^+^EPCs in the HCC microenvironment and BM displayed increased myeloid cell morphologies compared with those in the circulation and spleen of individuals with HCC (Figure 1A-B, Figure S1-2).

Human CD45^+^EPCs expressed MDSC surface markers, including CD11b, CD14, and the specific surface marker of MDSCs, LOX-1 18, 19. Compared with those from healthy donors, CD45^+^EPCs from patients with HCC, especially those from tumor tissues, expressed much higher levels of MDSC surface markers (Figure 1C). Consistently, CD45^+^EPCs from tumor-bearing mice, especially those from tumor tissues, also express higher levels of MDSC markers 19 including CD11b, Ly6C, and Ly6G, than those from tumor-free mice. Notably, CD45^+^EPCs in the BM displayed the highest level of MDSC surface markers (Figure 1D).

CD45^-^EPCs were relatively more mature than CD45^+^EPCs, as illustrated by their morphology. However, the morphology differed between humans and mice. In humans, CD45^-^EPCs were mainly nucleated red cells, while those from mice maintained the nucleus (Figure 1A-B, S1). Mouse CD45^-^EPCs from the spleen of tumor-free mice and tumor-bearing mice expressed Ly6G instead of CD11b and Ly6C; however, those from tumor tissues expressed low levels of MDSC surface markers (Figure 1D). Consistent with the relative maturity indicated by their morphology, RNA-seq revealed that CD45^-^EPCs lost the expression of most genes compared with CD45^+^EPCs (Figure 1E).

CD45^+^EPCs exert an immunosuppressive function by generating transforming growth factor (TGF)-β, IL-10, and reactive oxygen species (ROS) 7, 13. In the present study, human CD45^+^EPCs from the HCC microenvironment expressed higher levels of TGF-β than those in the circulation (Figure 1F, Figure S3A, Figure S4), and showed increased activation of neutrophil-related pathways, cell adhesion, and RNA regulation (Figure 1G, Figure S3B). Similarly, the CD45^+^EPCs from the tumor tissue of mice expressed activated myeloid cell-related pathway (Figure S3).

CD45^+^EPCs from tumor tissues displayed lower expression levels of erythroid cell development-related genes and higher levels of myeloid cell development-related genes (Figure S5). An *in vitro* erythroid cell development culture system revealed that CD45^+^EPCs from the BM and tumor tissue of tumor-bearing mice were not as efficient in generating mature red blood cells as CD45^+^EPCs from the BM of tumor-free mice (Figure 1H).

Above all, the EPCs, mainly CD45^+^EPCs, in HCC tissues displayed increased features of myeloid cells, including morphology, surface markers, TGF-β generation, and gene expression, compared with those from the circulation. Thus, CD45^+^EPCs, especially those in the tumor microenvironment (TME), comprise a high proportion of EDMCs.

### CD45^+^EPCs were chemoattracted by HCC macrophages mainly through CCR2

**Figure 2:**
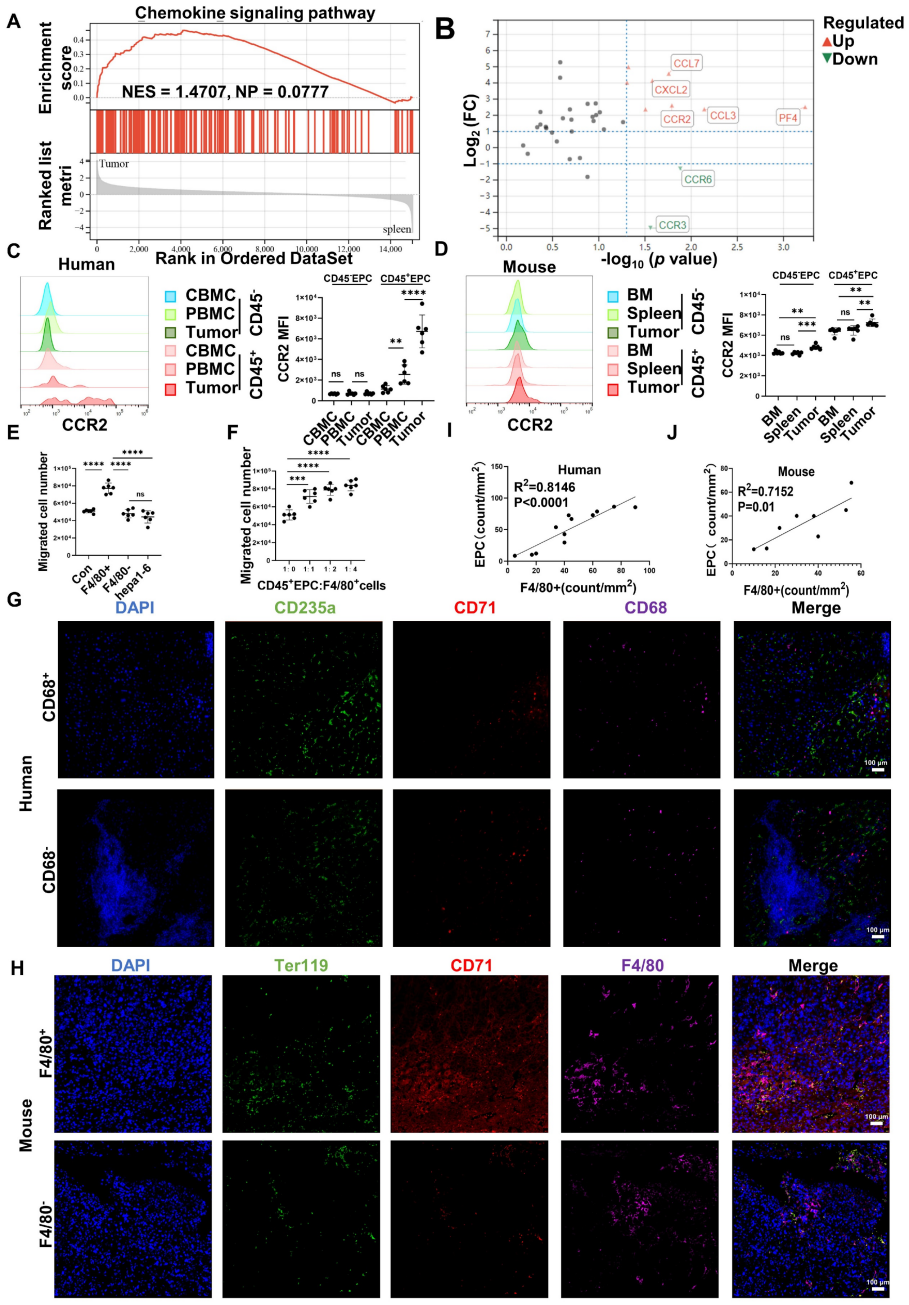
** CD45^+^ erythroid progenitor cells (EPCs) were chemoattracted by hepatocellular carcinoma (HCC) macrophages through C-C chemokine receptor type 2 (CCR2). (A)** Gene set enrichment analysis of the chemokine signaling pathway among CD45^+^EPCs from spleen and tumor tissues of tumor-bearing mice. **(B)** Volcano plots displaying differentially expressed genes associated with chemokine signals. Red dots represent the upregulated expressed transcripts (p < 0.05, log_2_ fold change>1), and green dots represent the downregulated expressed transcripts (p < 0.05, log_2_ fold change>1). **(C)** Representative and statistical analysis of the cumulative fluorescence intensity (MFI) of CCR2 on CD45^-^EPCs and CD45^+^EPCs from cord blood mononuclear cells (CBMCs), peripheral blood mononuclear cells (PBMCs) (n = 6), and tumor tissues (n = 6) from HCC patients. **(D)** Representative and statistical analysis of MFI of CCR2 on CD45^-^EPCs and CD45^+^EPCs from bone marrow, spleen and tumor tissues (n = 6) of orthotopic HCC tumor-bearing mice. **(E, F)** Cell migration assay. After 24 h of serum starving, CD45^+^EPCs from spleen of tumor-bearing mice (5×10^5^/mL) were suspended in serum-free medium and seeded in the upper chamber. Media containing 10% FBS (v/v) was placed into the lower chamber, as well as indicated cells in Matrigel matrix glue in indicated ratios. After 24 h, cells that had migrated into the medium of the low chamber were evaluated. Representative of one independent experiment, cumulative data are shown (n = 6). **(G-J)** Immunofluorescence staining of tumor. Paraffin tissue sections of human (CD71, red; CD235a, green; CD68, purple, DAPI, blue) and mouse (CD71, red; Ter119, green; F4/80, purple, DAPI, blue) HCC samples. Linear regression of the abundance of CD71^+^CD235a^+^ cells and macrophages was conducted. ns: non-significant ****p < 0.0001; ***p < 0.001; **p < 0.01.

Gene set enrichment analysis illustrated that tumor CD45^+^EPCs presented higher levels of chemokine signals than those in the spleen (Figure 2A). Among the chemokine receptors, CCR2 was found to be elevated in CD45^+^EPCs from the TME using RNA-seq (Figure 2B). Flow cytometry tests confirmed the high expression of CCR2 in CD45^+^EPCs in the HCC microenvironment of humans and mice, as well as CD45^+^EPCs in mouse HCC tissues (Figure 2C-D, Figure S6). Then, the chemoattraction capability of HCC tissue macrophages, mononuclear cells from tumor tissue without macrophages, and tumor cells to CD45^+^EPCs was tested using Transwell analysis. As a result, F4/80^+^ cells from HCC tissues substantially induced the migration of CD45^+^EPCs in a dose-dependent manner (Figure 2E-F). The association between EPCs (CD71^+^CD235^+^ cells for humans, CD71^+^Ter119^+^ for mice) and macrophages (CD68^+^ for humans, F4/80^+^ for mice) was analyzed in the tumor tissues using immunofluorescence analyses, which revealed that these two cell types were positively associated both in location and abundance (Figure 2G-J). Thus, EDMCs were chemoattracted by HCC macrophages mainly through CCR2 from EPCs in the circulation.

### CD45^+^EPCs compromised VECs, promoted local coagulation and HCC cell migration

**Figure 3:**
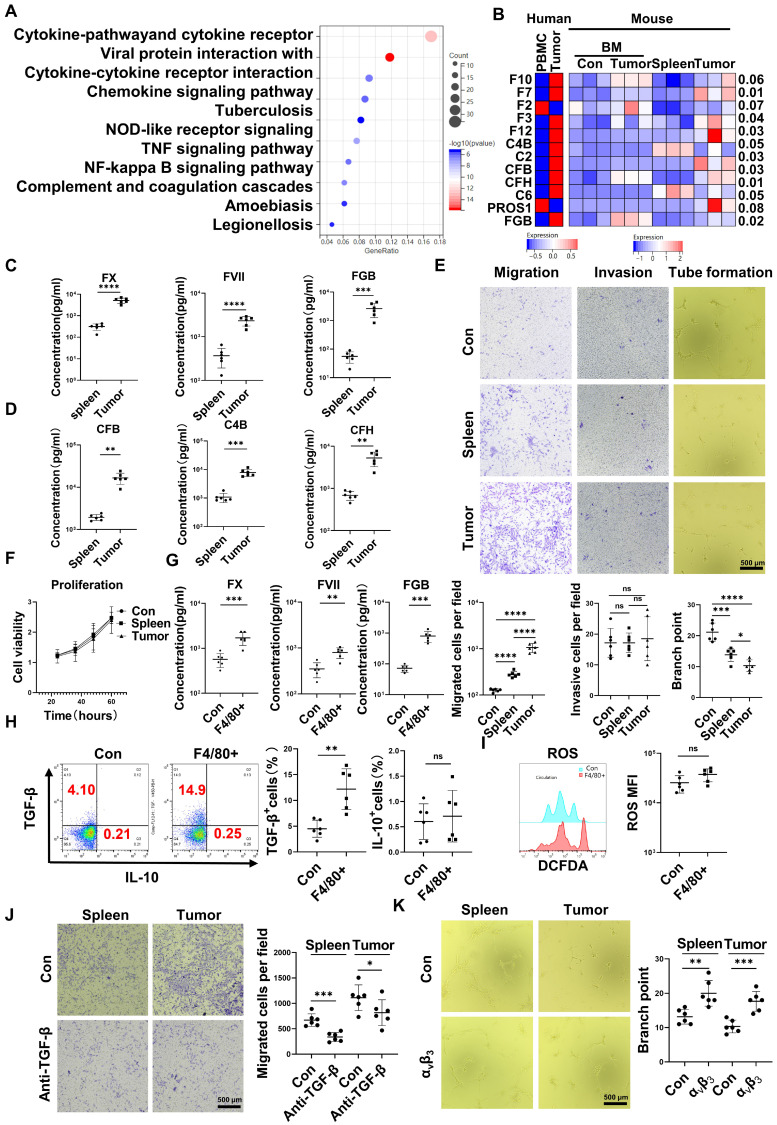
** CD45^+^ erythroid progenitor cells (EPCs) compromised vascular endothelial cells and promoted local coagulation. (A)** Kyoto Encyclopedia of Genes and Genomes pathway analysis of CD45^+^EPCs from spleen and tumor tissue of hepatocellular carcinoma (HCC) tumor-bearing mice. **(B)** Expression of complement- and coagulation-related genes in CD45^+^EPCs from peripheral blood mononuclear cells (PBMCs) and tumor tissue from one large tumor HCC patient, bone marrow from three tumor-free mouse and three tumor-bearing mouse, and spleen and orthotopic HCC tissue from three tumor-bearing mice, illustrated by RNA-seq. **(C, D)** ELISAs of FX, FVII, fibrinogen β chain (FGB), complement factor B (CFB), C4b, and complement factor H (CFH), in the conditioned media of CD45^+^EPCs from spleen and HCC tissue of tumor-bearing mice. **(E)** Cell migration and invasion assay: After 24 h of serum starving, 2×10^4^ endothelial cells (ECs) were suspended in serum-free medium and seeded in the upper chamber. Media containing 20% (v/v) FBS was placed into the lower chamber, as well as CD45^+^EPCs from the spleen of tumor-free mice, and the spleen and tumor tissue of tumor-bearing mice. After 24 h (migration tests) or 72 h (invasion tests), cells adhered to the lower surface were evaluated. Tube formation assay: 1×10^5^ ECs were cultured with conditioned medium of CD45^+^EPC from the spleen of tumor-free mice and the spleen and tumor tissue of tumor-bearing mice for 12 h. Representative of one independent experiment, cumulative data are shown (n = 6). **(F)** Cell counting kit-8 (CCK8) analysis: ECs were cocultured with CD45^+^EPCs from above sources; 24, 36, 48, and 60 h post seeding, CD45^+^EPCs were washed and CCK8 analysis was conducted. Representative of one independent experiment, cumulative data are shown (n = 6). **(G)** ELISAs of FX, FVII, and FGB of the conditioned media of macrophage chemoattracted CD45^+^EPCs and CD45^+^EPC not being chemoattracted by macrophage. **(H, I)** Representative and statistical analysis of flow cytometric analysis of TGF-β, IL-10, and ROS on macrophage chemoattracted CD45^+^EPCs and CD45^+^EPCs not being chemoattracted by macrophage. **(J)** Neutralizing antibody of TGF-β (10 μg/mL) and CD45^+^EPCs from spleen or tumor tissue of tumor-bearing mice were administered into the lower chamber with ECs in the upper chamber. Migration of ECs was evaluated. **(K)** 1×10^5^ ECs were cultured with conditioned medium of CD45^+^EPC from the spleen and tumor tissue of tumor-bearing mice for 12 h. α_v_β_3_ (10 μg/mL) was added, and tube formation assay conducted. Representative of one independent experiment, cumulative data are shown (n = 6). ****p < 0.0001; ***p < 0.001; **p < 0.01; *p < 0.05. con: control; DCFDA: 2′,7′-dichlorofluorescein diacetate; IL: interleukin; ns: non-significant; ROS: reactive oxygen species; TNF: tumor necrosis factor; TGF: transforming growth factor.

RNA-seq results indicated that CD45^+^EPCs from the TME displayed activation of the complement and coagulation cascades (Figure 3A). Specifically, FX, C4B, FVII, FXII, C2, complement factor (CF) B, CFH, fibrinogen β chain (FGB), and PROS1 levels were higher in CD45^+^EPCs from the TME than those in the circulation (Figure 3B). Enzyme-linked immunosorbent assay (ELISA) results confirmed the elevation of FX, FVII, FGB, C4b, CFB, and CFH in the conditioned media of CD45^+^EPCs from the TME compared with those from the spleen (Figure 3C-D). TGF-β induces endothelial cell migration 20, while FGB (β43-63) has been reported to compromise tube formation in endothelial cells 21. Thus, we speculated that CD45^+^EPCs in the TME might promote PVTT by compromising VECs and promoting local coagulation. As a result, mouse CD45^+^EPCs from the HCC microenvironment promoted the migration and compromised tube formation of VECs (Figure 3E). However, they did not increase the invasion or proliferation of VECs (Figure 3F). CD45^+^EPCs that were chemoattracted by macrophages from the circulation displayed higher levels of FX, FVII, FGB, and TGF-β, but not IL-10 and ROS (Figure 3G-I). Anti-TGF-β ceased the VEC migration-inductive capability of CD45^+^EPCs (Figure 3J). α_v_β_3_ blocked the destructive ability of VECs to form tubes via CD45^+^EPCs (Figure 3K). Additionally, CD45^+^EPCs induced HCC cell migration without impacting invasion or proliferation (Figure S7).

### Upregulation of C/EBPβ and m^6^A methylation induced the generation of FX, FVII, FGB, and TGF-β in CD45^+^EPCs from the TME

**Figure 4:**
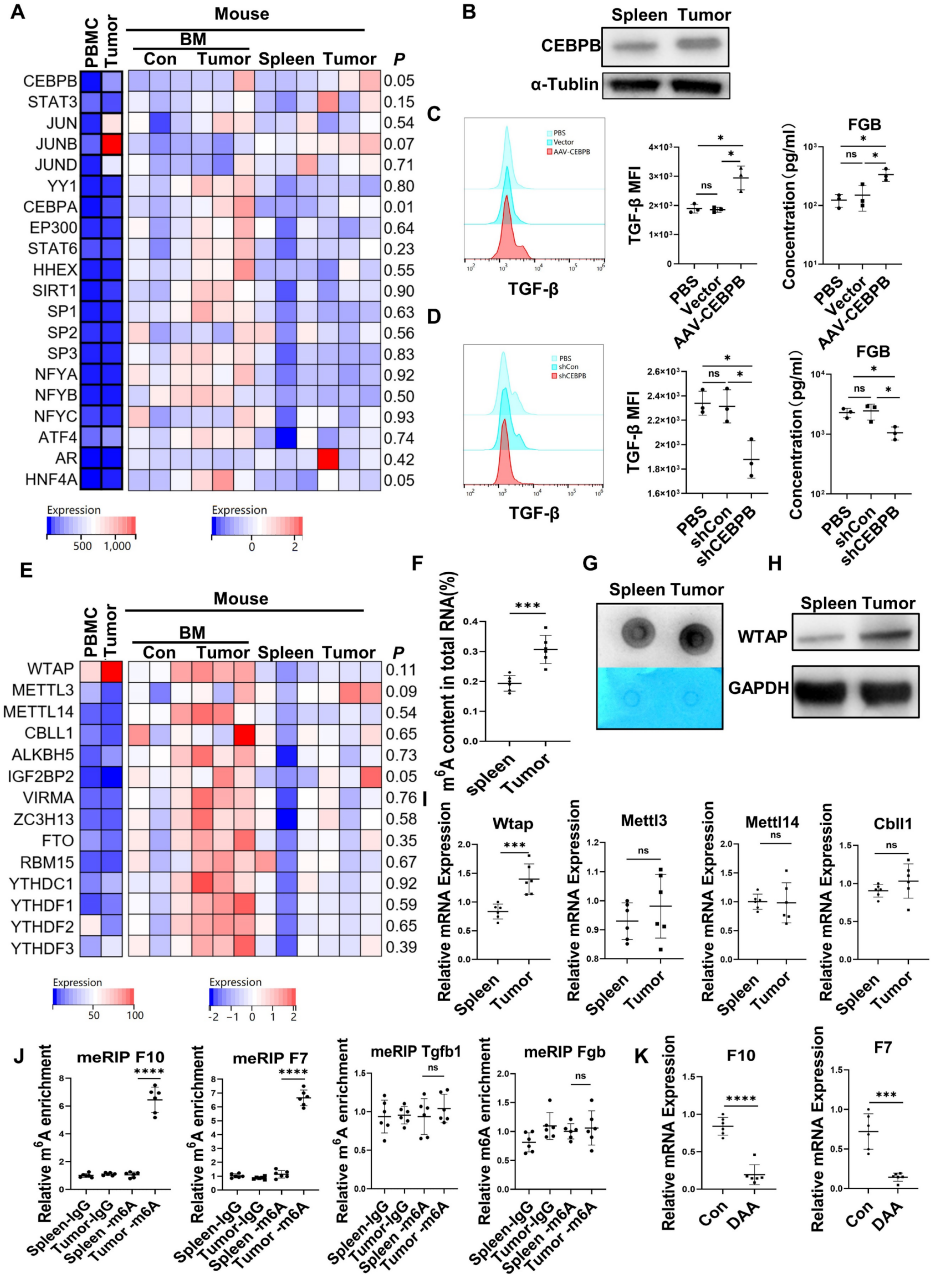
** Upregulation of CCAAT/enhancer binding protein beta (C/EBPβ) and m^6^A induced the generation of FX, FVII, fibrinogen β chain (FGB), and TGF-β of CD45^+^ erythroid progenitor cells (EPCs) in the tumor microenvironment. (A)** The expression of transcription factors of FX, FVII, FGB and TGF-β in CD45^+^EPCs from peripheral blood mononuclear cell (PBMC) and tumor tissue of one large tumor hepatocellular carcinoma (HCC) patient, bone marrow (BM) of three tumor-free mouse and three tumor-bearing mouse, and spleen and orthotopic HCC tissue of three tumor-bearing mice illustrated by RNA-seq. **(B)** Western blot of C/EBPβ in CD45^+^EPCs from spleen and HCC tissue of tumor-bearing mice. **(C)** AAV-CEBPB overexpression or AAV empty vectors were administered CD45^+^EPCs from the spleen of tumor-bearing mice. Intracellular TGF-β abundance was assessed using flow cytometry, while supernatant fibrinogen β chain (FGB) was quantified using ELISA. **(D)*** Cebpb* shRNA was administered to CD45^+^EPCs from tumor tissues and the abundances of TGF-β and FGB were evaluated. **(E)** Expression of m^6^A methylation-related genes in CD45^+^EPCs from PBMC and tumor tissue of one HCC patient, BM of three tumor-free mouse and three tumor-bearing mouse, and spleen and orthotopic HCC tissue of three tumor-bearing mice illustrated by RNA-seq. **(F, G)** m^6^A RNA methylation quantification (F) and m^6^A dot blot test (G) of CD45^+^EPCs from spleen and HCC tissue of tumor-bearing mice. **(H)** Western blot of Wtap of CD45^+^EPCs from spleen and HCC tissue of tumor-bearing mice. **(I)** qRT-PCR test of Wtap, Mettl3, Mettl14 and Cbll1 of CD45^+^EPCs from spleen and HCC tissue of tumor-bearing mice. **(J)** qRT-PCR of FX, FVII, Fgb, and Tgfb1 after MeRIP of CD45^+^EPCs from spleen and HCC tissue of tumor-bearing mice. **(K)** qRT-PCR of FX, FVII, Fgb, and Tgfb1 of CD45^+^EPCs from HCC tissues from tumor-bearing mice with or without 3-deazaadenosine (DAA) treatment. ns = non-significant. ****p < 0.0001; ***p < 0.001; *p < 0.05. con: control; Ig: immunoglobulin; ns: non-significant; PBS: phosphate buffered saline; TGF: transforming growth factor.

The expression of the transcription factors for FX 22, FVII 23, FGB 24, 25 and TGF-β 26, 27 in the CD45^+^EPCs from the TME and circulation was analyzed with RNA-seq. The results showed that the transcription factors for FGB and TGF-β, C/EBPβ were elevated in EDMCs from the TME (Figure 4A), which was confirmed by western blotting (Figure 4B). Additionally, overexpression of *Cebpb* by AAV increased production of TGF-β and FGB by splenic CD45^+^EPCs in tumor-bearing mice (Figure 4C). Meanwhile, *Cebpb* silencing consistently reduced TGF-β and FGB production by CD45^+^EPCs in tumor tissues (Figure 4D). Indeed, C/EBPβ is a common transcription factor of FGB 28 and TGF-β 29, suggesting that the generation of FGB and TGF-β might result from C/EBPβ upregulation.

The mRNA levels of m^6^A methylation-related genes were also elevated in CD45^+^EPCs from the TME compared to in those from the circulation (Figure 4E). The EpiQuik m^6^A RNA Methylation Quantification Kit and m^6^A dot blot confirmed that the m^6^A methylation levels in CD45^+^EPCs from the TME were higher than in those from the spleen (Figure 4F, 4G). Upregulation of Wtap was confirmed using western blotting and qRT-PCR (Figure 4H, 4I). METLL3 is the key component for m^6^A methylation. The combination by WTAP of METLL3 in CD45^+^EPCs from tumor was higher than those from spleen (Figure S8). MeRIP qRT-PCR illustrated that m^6^A methylation of the mRNA of FX and FVII increased in CD45^+^EPCs from the HCC microenvironment, while that of FGB and TGF-β was unaltered (Figure 4J). The m^6^A methylation inhibitor, 3-deazaadenosine (DAA), inhibited the production of FX and FVII in CD45^+^EPCs from the TME (Figure 4K).

### Wtap stabilized FX and FVII mRNA and enhanced their nuclear export in CD45^+^EPCs from the TME

**Figure 5:**
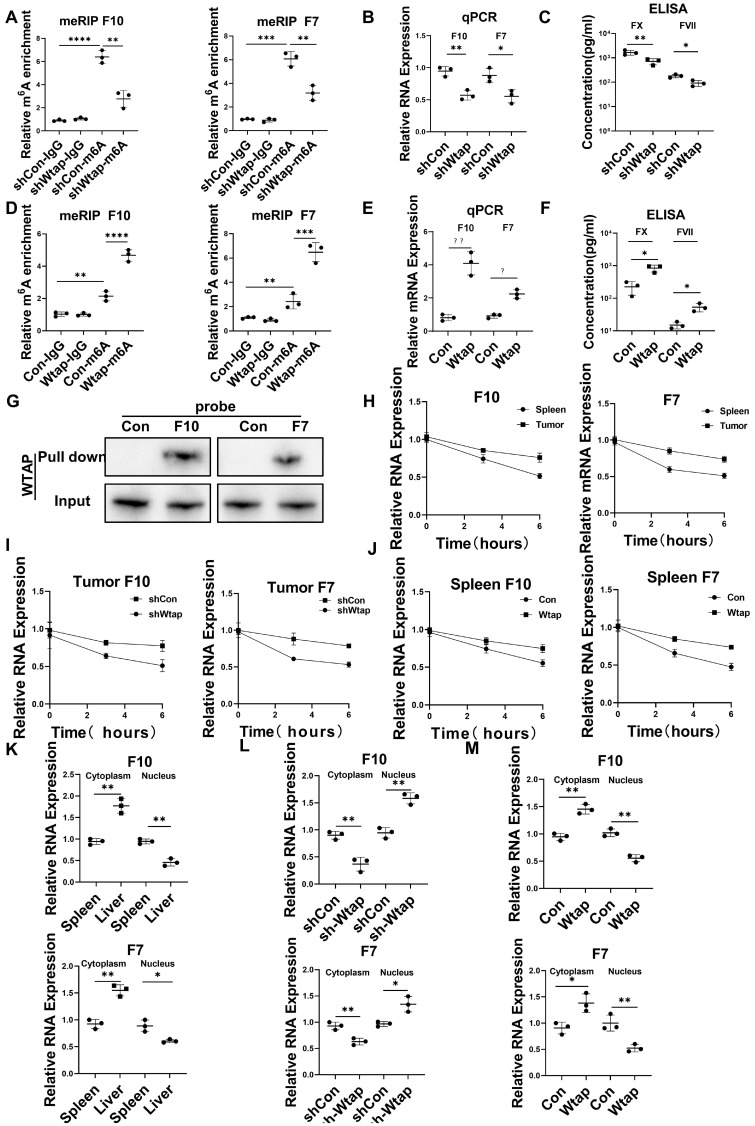
** Wtap stabilized the mRNA of F10 and F7 and enhanced their nuclear export in CD45^+^ erythroid progenitor cells (EPCs) from the tumor microenvironment. (A)** qRT-PCR of F10 and F7 after methylated RNA immune precipitation (MeRIP) in CD45^+^EPCs from mouse hepatocellular carcinoma (HCC) tissues pretreated with shRNA of Wtap. **(B, C)** qRT-PCR (B) and ELISA (C) of FX and FVII in CD45^+^EPCs from mouse HCC tissue pretreated with shRNA of Wtap. **(D)** qRT-PCR of FX and FVII after MeRIP in CD45^+^EPCs from the spleen of tumor-bearing mice overexpressed Wtap. **(E, F)** qRT-PCR (E) and ELISA (F) of FX and FVII in CD45^+^EPCs of tumor-bearing mice overexpressed Wtap. **(G)** Validation of FX and FVII mRNA interaction with Wtap protein. **(H)** qRT-PCR of F10 and F7 in CD45^+^EPCs from spleen and HCC tissue of tumor-bearing mice under the treatment of actinomycin D at different times. **(I)** qRT-PCR test of F10 and F7 in CD45^+^EPCs from tumor tissue pretreated with shRNA of Wtap under the treatment of actinomycin D at different times.** (J)** qRT-PCR of FX and FVII in CD45^+^EPCs from spleen of tumor-bearing mice pretreated with shRNA of Wtap under the treatment of actinomycin D at different times. **(K)** qRT-PCR of FX and FVII in the nucleus and cytoplasm of CD45^+^EPCs from spleen and HCC tissue of tumor-bearing mice. **(L)** qRT-PCR of FX and FVII in the nucleus and cytoplasm of the CD45^+^EPCs from tumor tissue pretreated with shRNA of Wtap. **(M)** qRT-PCR test of FX and FVII in the nucleus and cytoplasm of the CD45^+^EPCs from spleen of tumor-bearing mice pretreated with AAV of Wtap. ns = non-significant. ****p < 0.0001; ***p < 0.001; **p < 0.01; *p < 0.05. Con: control; ELISA: enzyme-linked immunosorbent assay; Ig: immunoglobulin; ns: non-significant; qPCR: quantitative polymerase chain reaction.

Silencing Wtap expression in CD45^+^EPCs from the TME using short hairpin RNA (shRNA) suppressed m^6^A methylation of FX and FVII mRNA (Figure 5A), as well as expression of their mRNA (Figure 5B) and protein (Figure 5C). Overexpression of Wtap by adeno-associated virus vectors of CD45^+^EPCs from the spleen of tumor-bearing mice enhanced m^6^A methylation of FX and FVII mRNA (Figure 5D), as well as expression of their mRNA (Figure 5E) and protein (Figure 5F). RNA pulldown results indicated that FX and FVII mRNA combined with the Wtap protein (Figure 5G). Actinomycin D treatment prolonged the lifetime of FX and FVII mRNA in CD45^+^EPCs from the TME compared to in those from the spleen of tumor-bearing mice (Figure 5H). Wtap shRNA inhibited the stability of FX and FVII mRNA in CD45^+^EPCs in the TME and Wtap overexpression enhanced mRNA stability (Figure 5I, 5J).

The presence of FX and FVII mRNA in the nucleus was lower in CD45^+^EPCs from the TME than that in the those from the spleen of tumor-bearing mice. Consistently, the presence of FX and FVII mRNA in the cytoplasm was higher in CD45^+^EPCs from the TME than in those from the spleen of tumor-bearing mice (Figure 5K). Silencing Wtap expression in CD45^+^EPCs from the TME terminated this phenomenon (Figure 5L, Table S4). Meanwhile, Wtap overexpression in CD45^+^EPCs from the spleen of tumor-bearing mice induced it (Figure 5M).

### CD45^+^EPCs from the TME promoted PVTT and were associated with an inferior prognosis

**Figure 6:**
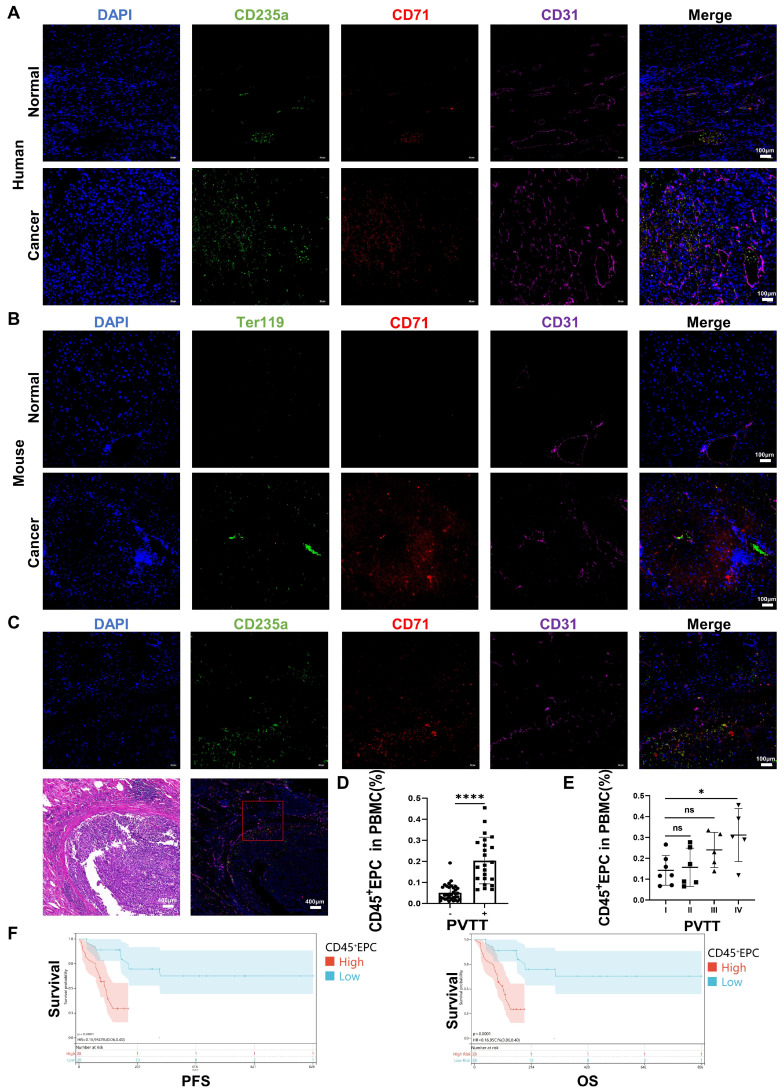
** CD45^+^ erythroid progenitor cells (EPCs) from the tumor microenvironment were positively associated with portal vein tumor thrombus (PVTT) and a worse prognosis. (A, B)** Immunofluorescence staining of CD71 (red), CD235a or Ter119 (green), CD31 (purple), and DAPI (blue) of paraffin-embedded human (A) and mouse (B) tissue sections. **(C)** Immunofluorescence staining of CD71 (red), CD235a (green), CD31 (purple), and DAPI (blue) of paraffin-embedded tissue sections of hepatocellular carcinoma (HCC) patients with PVTT. **(D)** CD45^+^EPCs in the circulation of HCC patients with or without PVTT. **(E)** CD45^+^EPCs in the circulation of HCC patients with different types of PVTT.** (F)** Progression-free survival (PFS) and overall survival (OS) of advanced HCC patients with high and low CD45^+^EPCs in circulation. ****p < 0.0001; *p < 0.05. ns: non-significant; PBMC: peripheral blood mononuclear cell.

We speculated that EDMCs, chemoattracted by HCC macrophages from EPCs in the circulation, compromised VECs and promoted local coagulation. The immunofluorescence tests illustrated that EPCs (CD71^+^CD235^+^ for humans, CD71^+^Ter119^+^ for mice) were enriched in the blood vessels of the liver of individuals with HCC, especially in the TME. The enrichment of EPCs in the TME was accompanied by disordered blood vessels and endothelium, especially in human HCC samples (Figure 6A, 6B). In addition, EPCs were enriched around the blood vessels with PVTT, where the endothelium was destroyed by VECs migrating into tumor tissues (Figure 6C). The association of CD45^+^EPCs in the circulation with PVTT indicated that patients with PVTT presented higher levels of CD45^+^EPCs (Figure 6D). In addition, the severity of PVTT was positively associated with the number of CD45^+^EPCs in the circulation (Figure 6E).

The prognostic value of CD45^+^EPCs in the circulation in patients with advanced HCC was investigated in terms of progression-free survival (PFS) and overall survival (OS) (Table S3). Discretization of the number of CD45^+^EPCs, which was a continuous variable, was performed using maximally selected rank statistics (max-stat) to determine the optimal cutoff point 30 for PFS and OS. The patients were divided into two groups according to the amount of CD45^+^EPCs in the circulation. Patients with low levels of CD45^+^EPCs demonstrated notably high PFS and OS rates (Figure 6F).

### CD45^+^EPCs in the TME promoted microvascular invasion through C/EBPβ and m6A methylation

**Figure 7:**
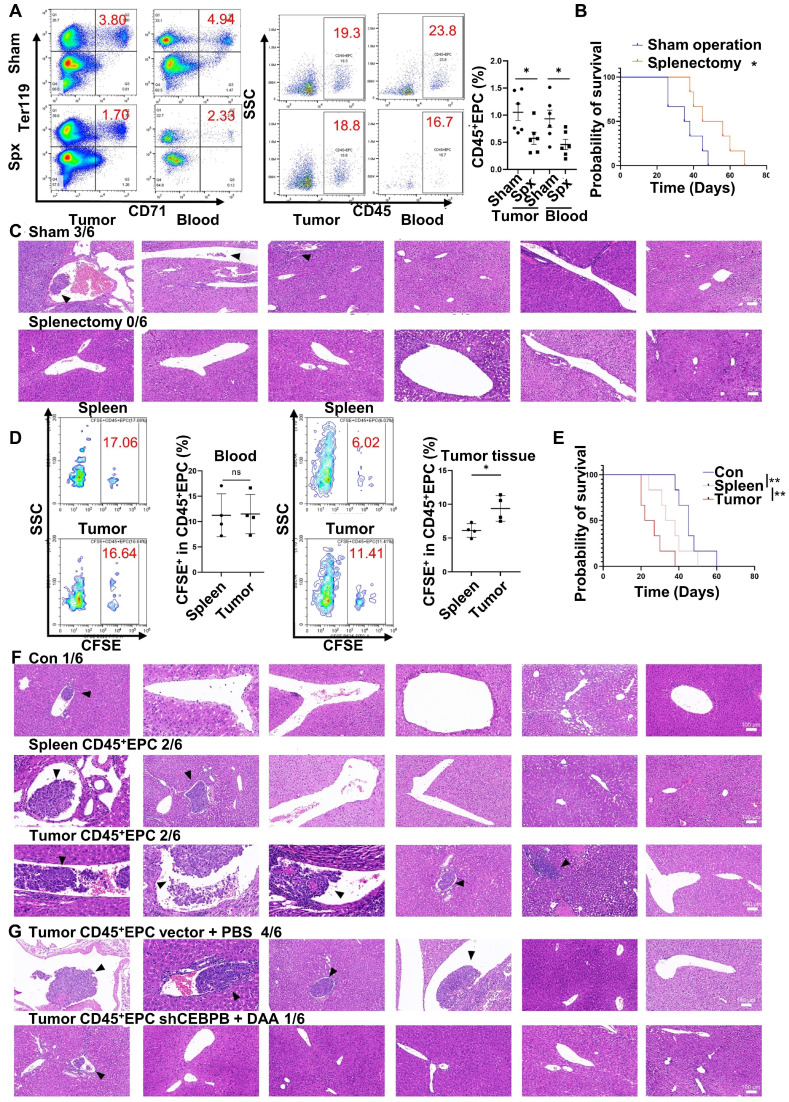
** CD45^+^ erythroid progenitor cells (EPCs) in the tumor microenvironment promoted microvascular invasion (MVI) through C/EBPβ and m6A methylation. (A-C)** Splenectomy or sham operation was conducted before construction of the orthotopic hepatocellular carcinoma (HCC) mouse model. The proportion of CD45^+^EPCs was assessed in tumor tissues and circulation (A). Survival curves were calculated with a humane endpoint, i.e., 10% body weight loss (B). MVI in peritumor areas (C). **(D-F)** CFSE-labeled CD45^+^EPCs were intravenously transferred from HCC tissues or the spleen to HCC-bearing splenectomized mice. (D) Analysis of CFSE^+^ cells in CD45^+^EPCs in circulation and HCC tissues. (E) Survival curves. (F) MVI analysis. **(G)** MVI analysis following the transfer of *Cebpb* shRNA and 3-deazaadenosine (DAA)-treated CD45^+^EPCs from HCC tissues to tumor-bearing splenectomized mice. shRNA-vector and PBS-treated CD45^+^EPCs from HCC tissues were used as the control. **p < 0.01; *p < 0.05. CFSE: Carboxyfluorescein Succinimidyl Ester; con: control; ns: non-significant; SSC: side scatter.

Extramedullary hematopoiesis in the spleen has been reported as the source of CD45^+^EPCs. Meanwhile, splenectomy reduces the abundance of circulating CD45^+^EPCs 7, 9. Similarly, in the present study, splenectomy reduced the abundance of CD45^+^EPCs in HCC tissues (Figure 7A) and improved survival (Figure 7B). Additionally, the incidence of microvascular invasion (MVI) was reduced following splenectomy (*p*=0.0455; Figure 7C). Subsequent intravenous (i.v.) transfer of CFSE-labelled CD45^+^EPCs from HCC tissue or the spleen to HCC-bearing splenectomized mice resulted in HCC tissue-derived CD45^+^EPCs being chemoattracted to the TME compared with those from the spleen (Figure 7D). Moreover, transfer of CD45^+^EPCs from HCC tissue decreased survival (Figure 7E) and increased the incidence of MVI (*p* = 0.02092) in tumor-bearing mice; this effect was not observed following transfer of CD45^+^EPCs from the spleen (*p* = 0.505; Figure 7F). To confirm the role of C/EBPβ and m^6^A methylation in the induction of EDMCs, *Cebpb*-silenced (Table S4) and DAA-treated CD45^+^EPCs from HCC tissues were transferred to tumor-bearing splenectomized mice. Consequently, MVI was reduced (Figure 7G). Collectively, these findings suggest that CD45^+^EPCs in TMEs promote MVI through C/EBPβ and m^6^A methylation.

## Discussion

PVTT reduces the possibility for radical surgery in patients with small tumors, limiting the application of interventional therapies such as transhepatic artery chemoembolization, reduction of treatment tolerance, and deterioration of liver functional reserve, thereby promoting the spread of tumors in the liver parenchyma and postoperative tumor recurrence 1, 31, 32. However, the mechanism of PVTT remains unclear 4, 6. A previous study found that IL-8 produced by tumor cells increased the permeability of VECs and adhesion between VECs and tumor cells 4. Portal vein serum promotes the metastatic potential of HCC cells 6; however, the specific mechanisms involved are unclear. The mechanism of PVTT includes the destruction of the vascular endothelium, tumor metastasis into the portal vein, and activation of coagulation. In the present study, we found that EDMCs accounted for a vital proportion of CD45^+^EPCs in the HCC microenvironment and were chemoattracted by HCC macrophages to the liver 17. EDMCs compromised VECs by inducing their migration via TGF-β expression and prevented their tube formation by β43-63 via FGB. EDMCs secrete FX and FVII, which might promote local coagulation. In addition, EDMCs might induce HCC cell migration to vessels. EDMCs and CD45^+^EPCs have been reported to be immunosuppressive and tumor-promoting 13, 17. Thus, HCC macrophage-induced EDMCs might be the specific mechanism underlying the high incidence of PVTT in HCC.

According to Long et al. 17, a certain proportion of CD45^+^EPCs are EDMCs, confirmed by the expression of surface markers and immunosuppressive activators of MDSCs. The present study showed that an increased proportion of CD45^+^EPCs met the definition of EDMCs in the HCC microenvironment. Remarkably, CD45^-^EPCs in individuals with tumors also displayed MDSC morphology and surface markers, which could allow them to be defined as EDMCs as well. However, the conformity of CD45^-^EPCs to the nomination of EDMCs was lower than that of CD45^+^EPCs. RNA sequencing and morphology analysis illustrated that CD45^-^EPCs were relatively more mature than CD45^+^EPCs. However, they might not develop from CD45^+^EPCs as tumor-associated CD45^+^EPCs rarely develop to nucleate red cells under the induction of erythropoietin, especially those from the TME. A previous study found that EDMCs consisted of CD45^+^EPCs 17; thus, we speculated that EDMCs developed from EPCs in patients with HCC.

The EDMCs in the HCC TME were induced and chemoattracted from circulating CCR2^+^CD45^+^EPCs by macrophages in the tumor tissue. The ligand of CCR2 is CCL2, which, within normal tissues and cell lines, is primarily expressed by aminocytes and islets of Langerhans 33. However, it can also be expressed by tumor-associated macrophages and Kupffer cells 34-36. Thus, we speculate that macrophages expressing CCL2 in HCC tissue act as a chemoattractant for CCR2^+^CD45^+^EPCs. However, CCL2/CCR2 axis might not be the only mechanism. Moreover, Transwell assay results revealed that circulating CD45^+^EPCs that were chemoattracted to macrophages expressed higher levels of FX, FVII, FGB, and TGF-β. Thus, we posit that macrophages in HCC tissue induce the differentiation of circulating CD45^+^EPCs into EDMCs.

Previous studies have confirmed the immunosuppressive capability of CD45^+^EPCs and EDMCs from the circulation and TME 7, 13, 17. However, their role in tumor development is not fully understood. MDSCs have been reported to promote tumor metastasis through multiple mechanisms other than their immune suppressive function 37. Similarly, the present study demonstrated the PVTT promotive capability of EDMCs. EPCs were enriched in blood vessels, especially in the TME, and were accompanied by disordered blood vessels and endothelium. In addition, EPCs were enriched around the blood vessels in PVTT, where the endothelium was destroyed and VECs migrated into tumor tissues. Mechanistically, CD45^+^EPCs in the HCC microenvironment induced VEC migration by TGF-β and compromised VEC tube formation by CFH. In addition, CD45^+^EPCs in the HCC microenvironment secrete FX and FVII, which may promote local coagulation. CD45^+^EPCs in circulation produce an unfavorable prognosis in patients with HCC. Thus, EDMC enrichment in the HCC microenvironment may be the specific mechanism underlying the high incidence of PVTT.

The RNA-seq results illustrated a critical difference between CD45^+^EPCs in circulation and those in the HCC microenvironment, which is a feature of EDMCs. Elevation of m^6^A methylation modification-related genes is also a feature of CD45^+^EPCs in the TME, and m^6^A methylation in tumor tissues regulates MDSC infiltration and function. In addition, METTL3 has been reported to be involved in the differentiation and development of normal myeloid cells 38 and may be involved in the induction of tumor-related MDSCs 39; still, the underlying mechanism is unknown. Furthermore, WTAP is significantly upregulated in HCC and promotes HCC development 40. This study found that WTAP stabilized FX and FVII mRNA and enhanced their nuclear export in EDMCs from the TME.

EPCs preferentially exist in the spleen 7, 9, 17. However, the present study found that the proportions of EDMCs were higher in tumor tissues and BM, and lower in the circulation and spleen. Therefore, splenectomy does not eliminate CD45^+^EPCs 7, 9, 10. Thus, we speculated that EDMCs might have originated from the BM and spleen.

A limitation of this study was the inconsistent morphology of CD45^-^EPCs among patients with HCC and in HCC-bearing mice. The conformation to the definition of EDMCs in CD45^-^EPCs from tumor-bearing mice was higher than those in human patients with HCC. The role of CD45^-^EPCs in the development of PVTT has not been investigated; thus, further investigation is required in this field. In the future, the prognostic value of EPCs or EDMCs among patients with HCC requires further investigation using multi-center studies with larger sample sizes.

## Conclusions

A significant proportion of the CD45^+^EPCs enriched in the HCC TME are EDMCs, which are induced, and chemoattracted, by HCC macrophages from the circulation to the TME. Within the TME, EDMCs promote PVTT by compromising the blood vessel endothelium, aggravating coagulation, and promoting HCC cell migration.

## Supplementary Material

Supplementary figures and tables, materials and methods.

## Abbreviations

BM: bone marrow
CCR2: C-C chemokine receptor type 2
CF: complement factor
EDMCs: erythroid-differentiated myeloid cells
ELISA: enzyme-linked immunosorbent assay
EPCs: erythroid progenitor cells
FGB: fibrinogen β chain
HCC: hepatocellular carcinoma
HRP: horseradish peroxidase
MeRIP: methylated RNA immunoprecipitation
OS: overall survival
PVTT: portal vein tumor thrombus
PFS: progression-free survival
ROS: reactive oxygen species
qRT-PCR: real-time quantitative reverse transcription PCR
shRNA: short hairpin RNA
TGF: transforming growth factor
TME: tumor microenvironment
VECs: vessel endothelial cells
