# Supplementary material for: Erythroid-transdifferentiated myeloid cells promote portal vein tumor thrombus in hepatocellular carcinoma
Source: Theranostics. 2023 Jul 31;13(13):4316–32. doi: 10.7150/thno.82907 (PMC10465220; doi:10.7150/thno.82907)
Supplement: Supplementary file 1 — Supplementary figures and tables, materials and methods. [file thnov13p4316s1.pdf]

Supplementary materials

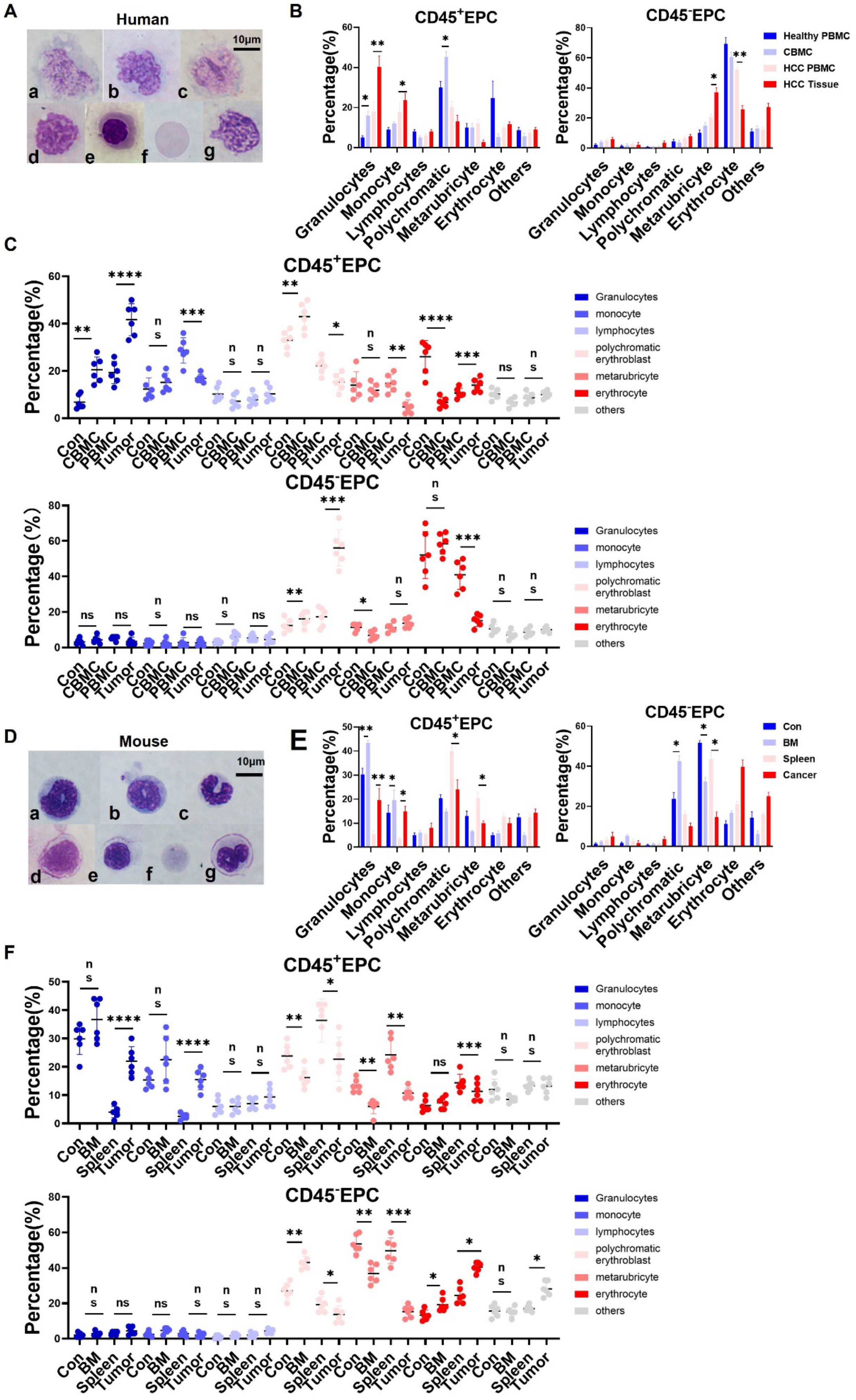

Figure S1. Morphology of erythroid progenitor cells (EPCs). (A) Typical morphology of granulocytes, lymphocytes, monocytes, polychromatic erythroblast, metarubricyte, erythrocyte, and atypical cells of human. **(B, C)** Statistical analysis of the proportion of granulocytes, lymphocytes, monocytes, polychromatic erythroblast, metarubricyte, erythrocyte, and atypical cells in $CD45^{+}$EPCs and $CD45^{-}$EPCs in the circulation of a healthy donor, cord blood of health infants, circulation of hepatocellular carcinoma (HCC) patients, and HCC tissues. **(D)** Typical morphology of granulocytes, lymphocytes, monocytes, polychromatic erythroblast, metarubricyte, erythrocyte, and atypical cells of mice. **(E, F)** Statistical analysis of the proportion of granulocytes, lymphocytes, monocytes, polychromatic erythroblast, metarubricyte, erythrocyte, and atypical cells in $CD45^{+}$EPCs and $CD45^{-}$EPCs in bone marrow (BM) of tumor-free mice and BM, spleen, and HCC tissues of tumor-bearing mice. ****$p < 0.0001$; ***$p < 0.001$; **$p < 0.01$; *$p < 0.05$. PBMC: peripheral blood mononuclear cell.

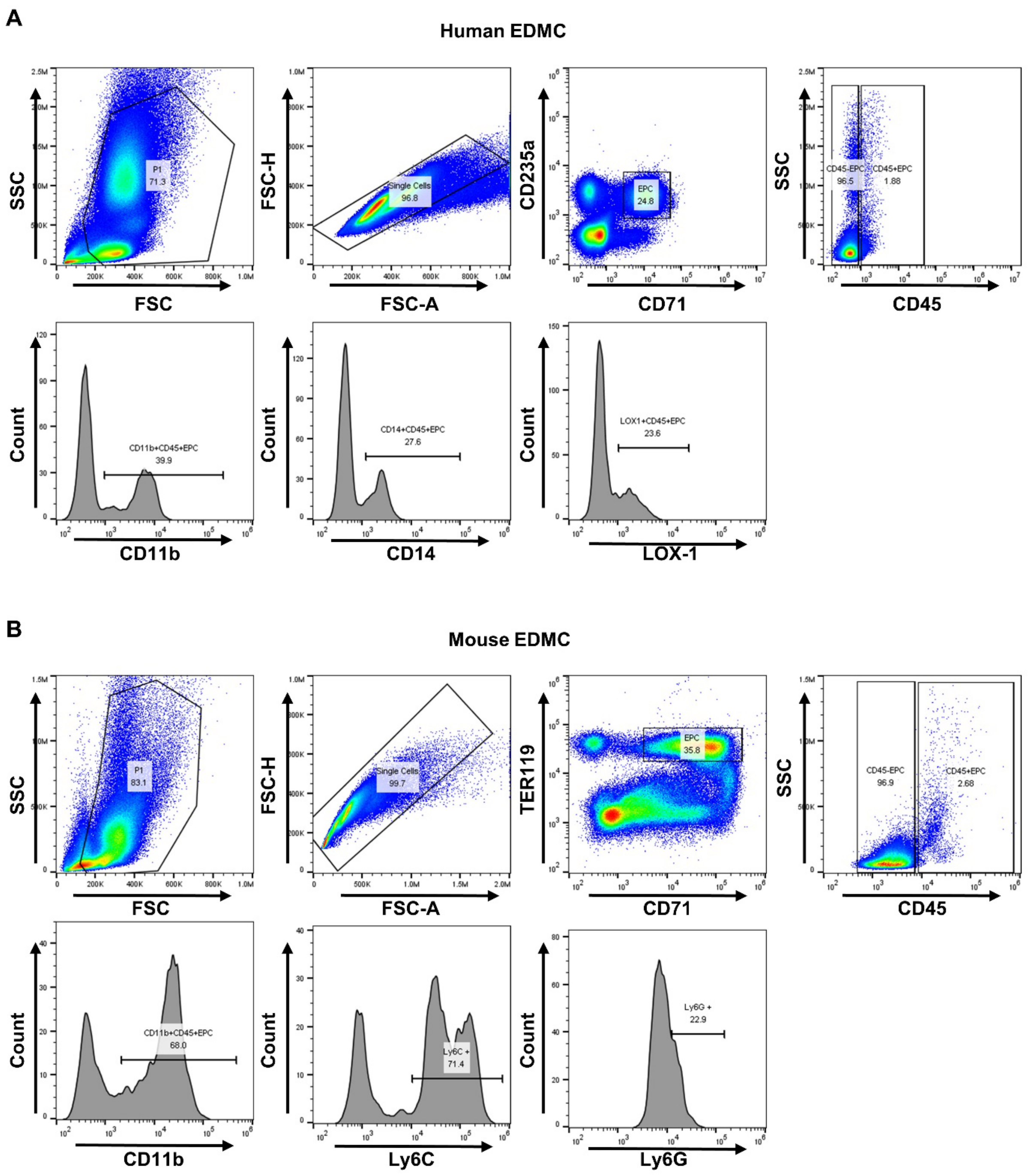

Figure S2. **Gating strategy for erythroid-transdifferentiated myeloid cells (EDMCs).**

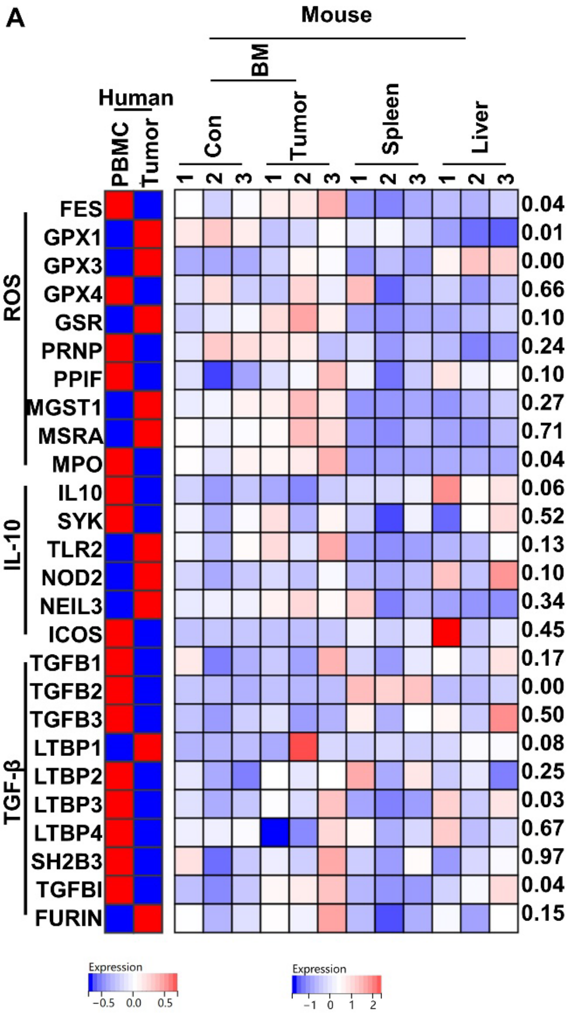

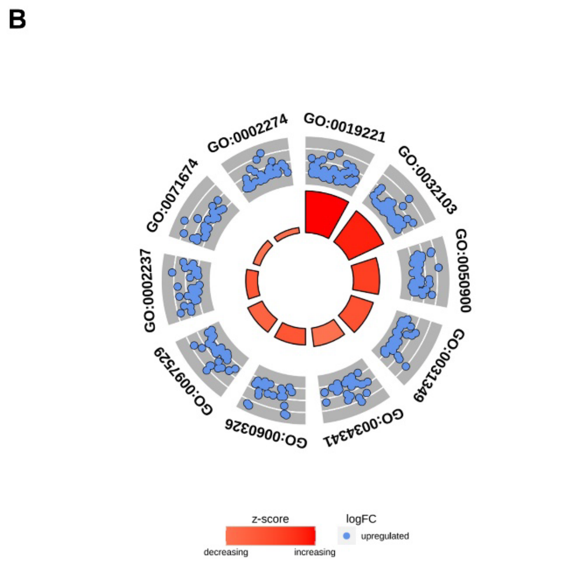

Mouse

ID	Description
GO:0019221	cytokine-mediated signaling pathway
GO:0032103	positive regulation of response to external stimulus
GO:0050900	leukocyte migration
GO:0031349	positive regulation of defense response
GO:0034341	response to interferon-gamma
GO:0060326	cell chemotaxis
GO:0097529	myeloid leukocyte migration
GO:0002237	response to molecule of bacterial origin
GO:0071674	mononuclear cell migration
GO:0002274	myeloid leukocyte activation

Figure S3. Whole-transcriptome analysis using RNA-seq on CD45$^+$ erythroid progenitor cells (EPCs) from circulation and tumor tissues. (A) Whole-transcriptome analysis using RNA-seq was conducted on CD45$^+$EPCs from peripheral blood mononuclear cell (PBMC) and tumor tissue of one hepatocellular carcinoma (HCC) patient, bone marrow of three tumor-free mouse and three tumor-bearing mouse, and spleen and orthotopic HCC tissue of three tumor-bearing mice. The expression of ROS-, IL-10- and TGF-β-related genes were analyzed. **(B)** Gene Ontology analysis of differential expressed genes between CD45$^+$EPCs from the spleen and orthotopic HCC tissue of tumor-bearing mice. IL: interleukin; ROS: reactive oxygen species; TGF: transforming growth factor

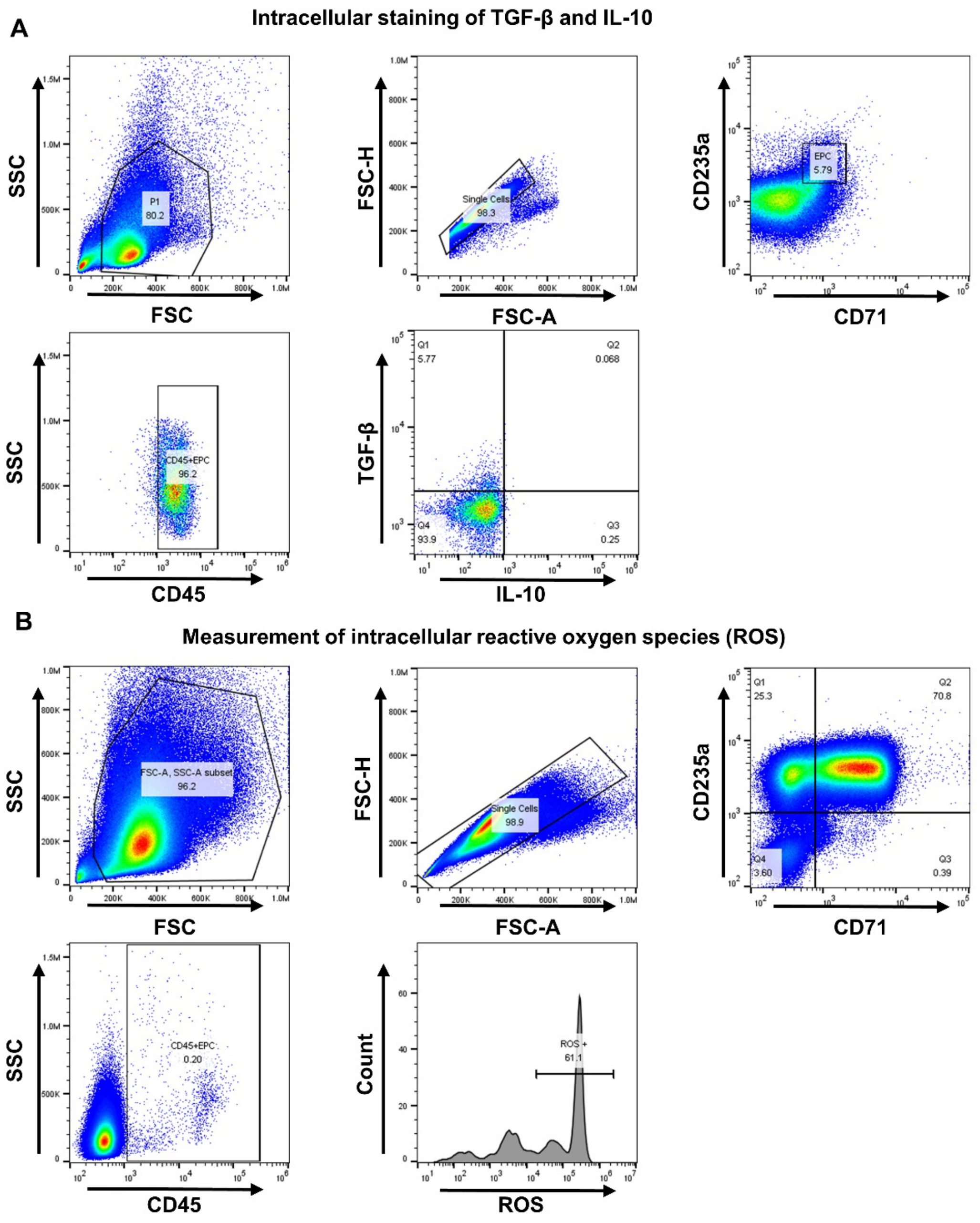

Figure S4. **Gating strategy for intracellular TGF-β, IL-10, and reactive oxygen species from CD45$^+$ erythroid progenitor cells.** IL: interleukin; TGF: transforming growth factor

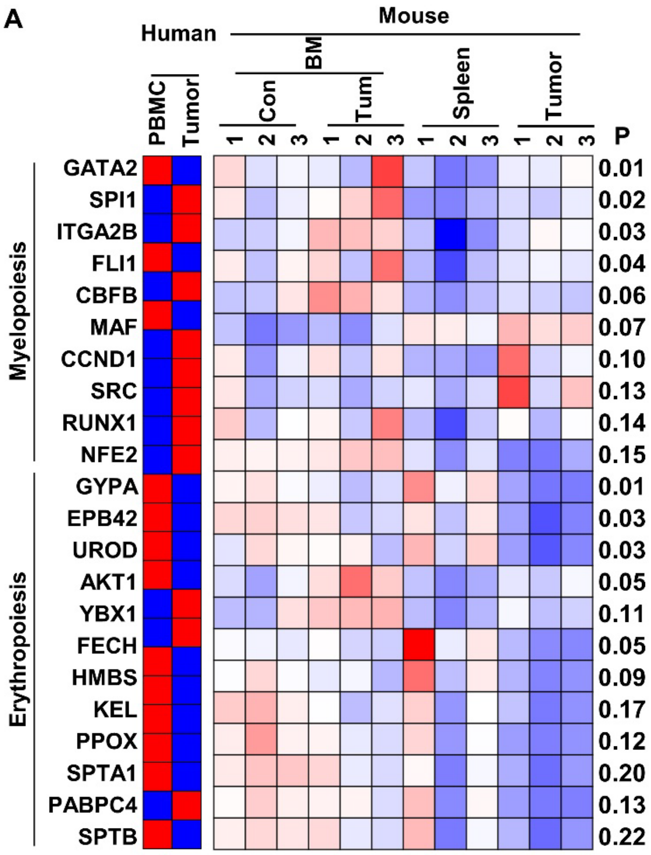

Figure S5. Whole-transcriptome analysis using RNA-seq. This analysis was conducted on $CD45^{+}$EPCs from peripheral blood mononuclear cell (PBMC) and tumor tissue of one hepatocellular carcinoma (HCC) patient, bone marrow of three tumor-free mouse and three tumor-bearing mouse, and spleen and orthotopic HCC tissues of three tumor-bearing mice. The expression of erythroid cell development related genes and myeloid cell development were analyzed. BM: bone marrow; con: control

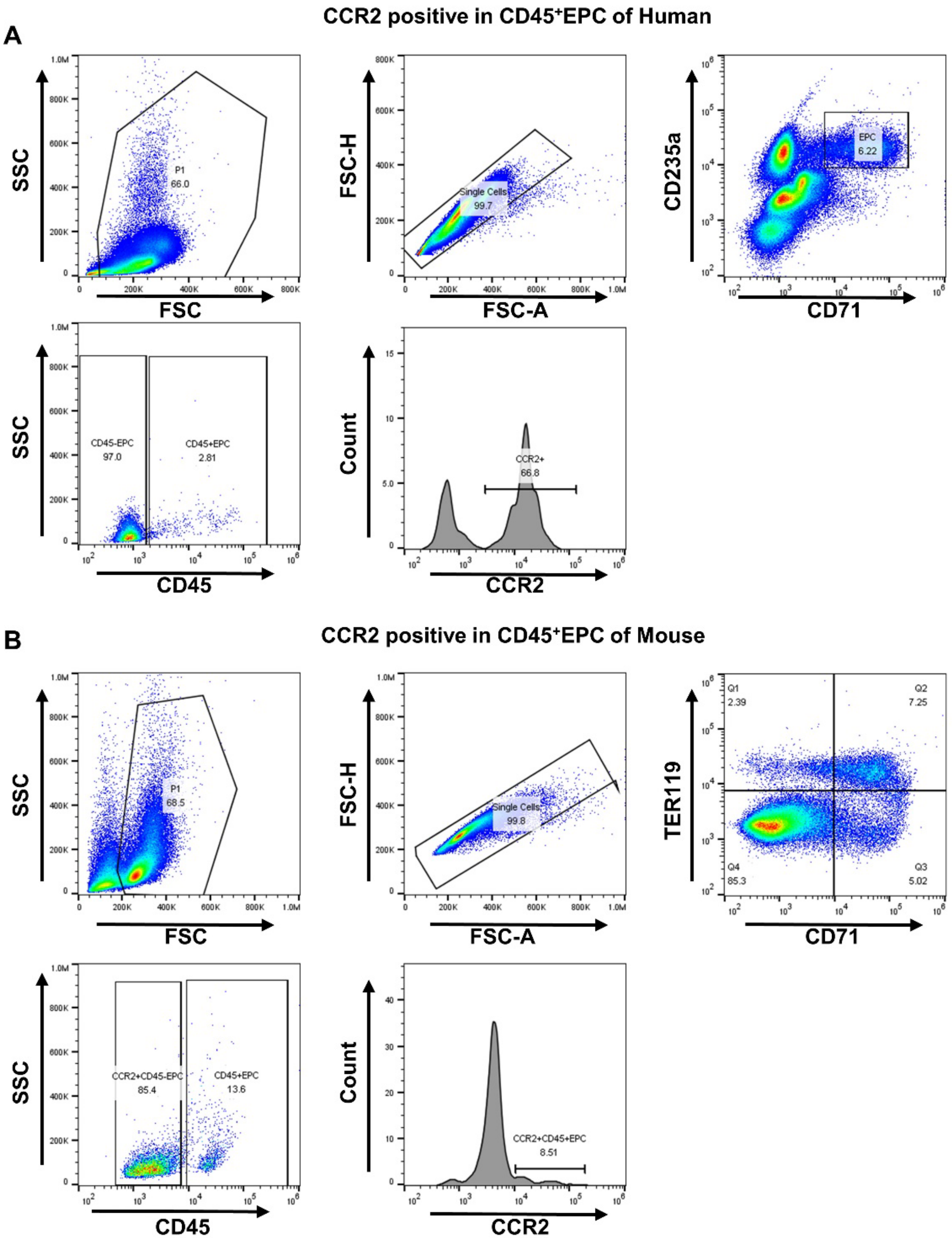

Figure S6. **Gating strategy for C-C chemokine receptor type 2 (CCR2)-positive cells in $CD45^+$ erythroid progenitor cells.**

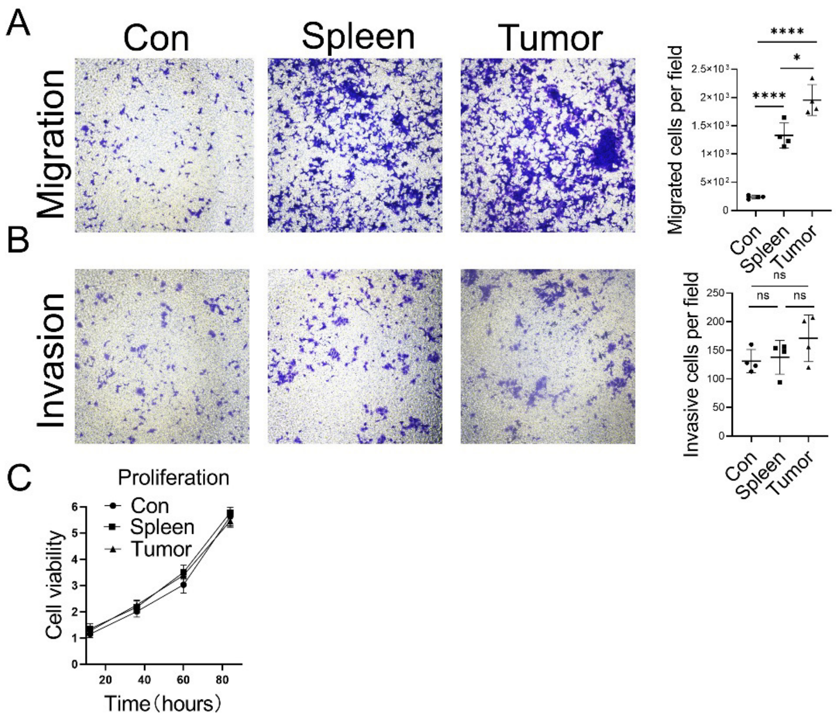

Figure S7. CD45⁺ erythroid progenitor cells (EPCs) induced migration of hepatocellular carcinoma (HCC) cells without impacting invasion or proliferation. After 24 h of serum starvation, HCC cells (5×10^4/mL) were suspended in serum-free media and seeded in the upper chamber. Media containing 20% FBS was placed in the lower chamber, as well as $CD45^+$EPCs (1×10^6/well) from HCC tumor tissue or the spleen of tumor-bearing mice. Cells adhered to the lower surface were evaluated. (A) Migration analysis after 24 h; (B) invasion analysis after 72 h. (n=4). (C) Proliferation curves of Hepa1-6 cells cocultured with $CD45^+$EPCs. ****$p < 0.0001$; *$p < 0.05$. Con: control

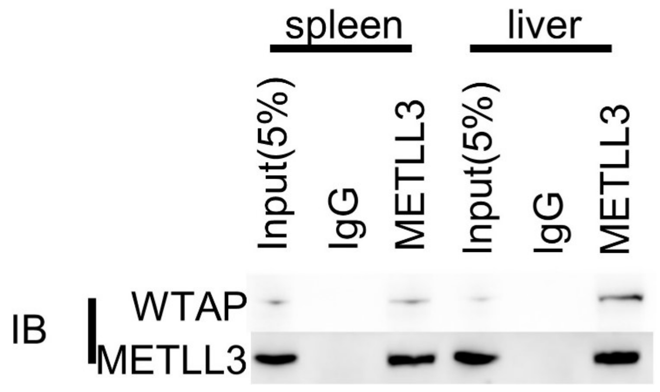

Figure S8. Co-IP assays of METLL3 and WTAP. Protein extraction of $CD45^+$ erythroid progenitor cells (EPCs) from spleen and tumor tissue of hepatocellular carcinoma (HCC) in situ mice models. Immunoprecipitated with anti-METLL3 antibody and detected by anti-WTAP and anti-METLL3. Input fractions represent 5% of total proteins in the Co-IP assays.

Table S1: Primers used in qRT-PCR.

Mouse primer name	Base sequence (5′ to 3′)
Tgfb1	ACTGGAGTTGTACGGCAGTG
	GGGGCTGATCCCGTTGATTT
Fgb	CAGGATGGGACCCACAGAAC
	GATTGGCTGCATGGCATCTG
Wtap	GAACCTCTTCCTAAAAAGGTCCG
	TTAACTCATCCCGTGCCATAAC
Mettl3	GGACTCTGGGCACTTGGATTTA
	CAGGTGCATCTGGCGTAGAG
Mettl14	CAGAGGCGGCTTTACTCCTC
	GCTGGGAGTGAGCTCTGAAG
Cbll1	CCGCAAGGTGATGAAGAAGG
	AGAGAACCTCGTGTGCACTG
F7	CAAGTACGACGGGTGACACA
	GGCACCACGTAGTCAGTGAA
F10	GTCCCCTGACCTCATTGCTG
	CACACTGGTCGCCGTCTTTAT
Gapdh	AACTTTGGCATTGTGGAAGG
	ACACATTGGGGGTAGGAACA

Table S2: Reagents and resources.

REAGENT or RESOURCE	SOURCE	IDENTIFIER
Antibodies		
Rabbit Loading Control Panel Kit	Affinity Biosciences, Jiangsu, China	Cat#KF007
CEBP beta antibody	Affinity Biosciences, Jiangsu, China	Cat#AF6202
WTAP antibody	Affinity Biosciences, Jiangsu, China	Cat#DF3282
Tubulin-alpha antibody	Affinity Biosciences, Jiangsu, China	Cat#AF7010
Brilliant Violet 605™ anti-human CCR2	BioLegend, San Diego, CA, USA	Cat#357213
Brilliant Violet 605™ anti-mouse CCR2	BioLegend, San Diego, CA, USA	Cat#150615
Goat anti-rabbit IgG H&L (HRP)	Abcam, Cambridge, MA, USA	Cat#ab6721
m6A antibody	Sysy, Göttingen, Germany	Cat#202003
GAPDH antibody	Affinity Biosciences, Jiangsu, China	Cat#T0004
PE/Cyanine7 anti-human CD14	BioLegend, San Diego, CA, USA	Cat#325617
Pacific Blue™ anti-human CD235a	BioLegend, San Diego, CA, USA	Cat#349108
PE anti-human LOX-1	BioLegend, San Diego, CA, USA	Cat#358604

FITC anti-human CD11b (ICRF44)	BioLegend, San Diego, CA, USA	Cat#35-0118-T100
Alexa Fluor® 700 anti-human CD15 (SSEA-1)	Tonbo Biosciences, San Diego, CA, USA	Cat#301920
ANTI-MO CD71 (R17217 (RI7 217.1.4)) SB436	BioLegend, San Diego, CA, USA	Cat#62-0711-82
FITC anti-human CD45 (HI30)	Invitrogen, Carlsbad, CA, USA	Cat#35-0459-T100
Anti-glycophorin A antibody [YTH89.1]	Abcam, Cambridge, MA, USA	Cat#ab33386
ANTI-HU CD71 OKT9 BIOTIN	Invitrogen, Carlsbad, CA, USA	Cat#13-0719-82
ANTI-MO CD71 R17217 BIOTIN	Invitrogen, Carlsbad, CA, USA	Cat#13-0711-82
violetFluor™ 450 anti-mouse F4/80 (BM8.1)	Tonbo Biosciences, San Diego, CA, USA	Cat#75-4801-U025
ANTI-HU CD235A HIR2 BIOTIN	Invitrogen, Carlsbad, CA, USA	Cat#13-9987-82
PE-Cyanine7 anti-mouse CD45 (30-F11)	Tonbo Biosciences, San Diego, CA, USA	Cat#60-0451-U100
Anti-CD31 antibody (RM1006)	Abcam, Cambridge, MA, USA	Cat#ab281583
ANTI-MO CD45 30-F11 BIOTIN	Invitrogen, Carlsbad, CA, USA	Cat#13-0451-82
PerCP/Cyanine5.5 anti-human CD71	BioLegend, San Diego, CA, USA	Cat#334114
PE goat anti-rat IgG (minimal x-reactivity)	BioLegend, San Diego, CA, USA	Cat#405406

Biotin anti-mouse F4/80	BioLegend, San Diego, CA, USA	Cat#123105
ANTI-HU CD14 61D3 BIOTIN	Invitrogen, Carlsbad, CA, USA	Cat#13-0149-82
ANTI-MO TER-119 BIOTIN	Invitrogen, Carlsbad, CA, USA	Cat#13-5921-85
Mounting medium with DAPI	Abcam, Cambridge, MA, USA	Cat#ab104139
Anti-F4/80 antibody (SP115)	Abcam, Cambridge, MA, USA	Cat#ab111101
Anti-CD68 antibody (C68/684)	Abcam, Cambridge, MA, USA	Cat#ab201340
PerCP-eFluor 710 anti-mouse CD71 (transferrin receptor) (R17217 (RI7 217.1.4))	Invitrogen/eBioscience, CA, USA	Cat#46-0711-82
FITC anti-mouse TER-119 (TER-119)	Tonbo Biosciences, San Diego, CA, USA	Cat#35-5921-U025
PEcy7 anti-mouse CD45 (30-F11)	Tonbo Biosciences, San Diego, CA, USA	Cat#60-0451-U100
APC anti-mouse CD71 (transferrin receptor) (R17217 (RI7 217.1.4))	Invitrogen/eBioscience, CA, USA	Cat#17-0711-82
PE anti-mouse TER-119 (TER-119)	Tonbo Biosciences, San Diego, CA, USA	Cat#50-5921-U100
FITC anti-human CD71 (transferrin receptor)	Invitrogen/eBioscience, CA, USA	Cat#11-0719-42
PE anti-human CD235a (HIR2)	Invitrogen/eBioscience, CA, USA	Cat#12-9987-82
APC anti-human CD45 (HI30)	Tonbo Biosciences, San Diego, CA, USA	Cat#20-0459-T100

PE/Cyanine7 anti-human CD45 (HI30)	BioLegend, San Diego, CA, USA	Cat#304015
BV421 anti-human TGF-β1 (TW4-9E7)	BD Bioscience, Franklin Lakes, NJ, USA	Cat#562962
PE/Cyanine7 anti-human IL-10 (jes3-9D7)	Invitrogen/eBioscience, CA, USA	Cat#25-7108-42
Anti-human CD14 (61D3), Alexa Fluor™ 700	Invitrogen/eBioscience, CA, USA	Cat#56-0149-42
Anti-human CD45	Abcam, Cambridge, MA, USA	Cat#ab8216
Anti-human CD71	Abcam, Cambridge, MA, USA	Cat#ab84036
Anti-human CD235	Abcam, Cambridge, MA, USA	Cat#ab33386
Anti-mouse CD45	Abcam, Cambridge, MA, USA	Cat#ab208022
Anti-mouse CD71	Abcam, Cambridge, MA, USA	Cat#ab269513
Anti-mouse TER 119	GeneTex, Irvine, CA, USA	Cat#GTX01475
Donkey anti-rat Alexa Fluor 488	Abcam, Cambridge, MA, USA	Cat#ab150153
Donkey anti-rabbit Alexa Fluor 594	Abcam, Cambridge, MA, USA	Cat#ab175692
Donkey anti-mouse Alexa Fluor 647	Abcam, Cambridge, MA, USA	Cat#ab150111
Goat anti-rat Alexa Fluor 488	Abcam, Cambridge, MA, USA	Cat#ab150165
Goat anti-rabbit Alexa Fluor 594	Abcam, Cambridge, MA, USA	Cat#ab150088
Goat anti-mouse Alexa Fluor 647	Abcam, Cambridge, MA, USA	Cat#ab150119
ELISA		
Mouse FX ELISA kit	Mlbio, Shanghai, China	Cat#ml001927
Mouse FVII ELISA kit	Mlbio, Shanghai, China	Cat#ml037570

Mouse FGB ELISA kit	Mlbio, Shanghai, China	Cat#ml900257V
Mouse C4b ELISA kit	Mlbio, Shanghai, China	Cat#ml058702V
Mouse CFB ELISA kit	Mlbio, Shanghai, China	Cat#ml555615V
Mouse CFH ELISA kit	Mlbio, Shanghai, China	Cat#ml602855V
Cytokine and neutralizing antibodies		
Recombinant human integrin alpha V beta 3	R&D Systems, Minneapolis, MN, USA	Cat#3050-AV-050
Recombinant mouse integrin alpha V beta 3	R&D Systems, Minneapolis, MN, USA	Cat#7889-AV-050
Magna RIP™ RNA binding Protein immunoprecipitation kit	Millipore, Billerica, MA, USA	Cat#17–700
MeRIP kits	PerxcyBiology, Guangzhou, China	Cat#PXM6A1
Recombinant human erythropoietin	Peprotech, Rocky Hill, NJ, USA	Cat#100-64
TGF beta-1,2,3 monoclonal antibody (1D11.16.8), functional grade	Invitrogen/eBioscience, CA, USA	Cat#16-9243-85
Mouse IgG1 kappa isotype Control (P3.6.2.8.1), functional grade	Invitrogen/eBioscience, CA, USA	Cat#16-4714-82
Others		
Cell Counting Kit-8	Dojindo, Kumamoto, Japan	Cat#CK04

Reactive oxygen species (ROS) Assay kit	Boxbio, Beijing, China	Cat#AKCE002-2
Intracellular fixation & Permeabilization BUFFER	Invitrogen/eBioscience, CA, USA	Cat#AKCE002-2
Cell stimulation cocktail	Invitrogen/eBioscience, CA, USA	Cat#00-4970-30
Protein Transport Inhibitor Cocktail	Invitrogen/eBioscience, CA, USA	Cat#00-4975
3-Deazaadenosine	APExBIO, Houston, TX, USA	Cat#B6121
Matrigel matrix glue	Corning, Corning, NY, USA	Cat#356234
Transwell Permeable Supports	Corning, NY, USA	Cat#3422
Fetal bovine serum	Cegrogen Biotech, Ebsdorfergrund, Germany	Cat#A0500-3010
Crystal violet	Solarbio Life Sciences, Beijing, China	Cat#G1062
Methylene blue staining solution (0.2%)	Solarbio Life Sciences, Beijing, China	Cat#G1301
Actinomycin D	Aladdin, Shanghai, China	Cat#A113142
Lymphoprep™	Stemcell, Vancouver, Canada	Cat#07801
Percoll	GE Healthcare, Chicago, IL, USA	Cat# 17089109-1
MagniSort™ streptavidin positive Selection beads	Invitrogen, Thermo Fisher Scientific, Waltham, USA	Cat#MSPB-6003
EpiQuik m6A RNA methylation kit	Epigentek, Farmingdale, NY, USA	Cat# EPT-P-9005-48

IMMOBILON WESTERN CHEMILUM HRP	Millipore, Billerica, MA, USA	Cat#638173

Table S3: Target sequences of *shWtap* and *shCebpb*.

Gene symbol	Target sequence (5′–3′)
shCebpb	ACAAGCTGAGCGACGAGTACA
shCebpb	GGAAAGTACACAGATCTTAAT
shWtap	GGAAAGTACACAGATCTTAAT
shWtap	GCACGGGATGAGTTAATTCTA

Table S4. Baseline demographic and clinical characteristics of hepatocellular carcinoma patients with high or low CD45$^+$ erythroid progenitor cells in circulation.

Characteristics	Level	Overall	CD45$^+$EPCs High	CD45$^+$EPCs Low	*p*
**n**		56	28	28	
**Age (mean, SD)**		55.929 (12.950)	57.179 (11.920)	54.679 (14.011)	0.4752
**Sex (n, %)**	Female	6 (10.71)	3 (10.71)	3 (10.71)	1
	Male	50 (89.29)	25 (89.29)	25 (89.29)	
**TB (median, IQR)**		21.050 (15.325, 45.175)	30.850 (15.498, 57.050)	18.600 (14.375, 37.700)	0.2316
**ALB (median, IQR)**		34.700 (30.875, 37.625)	32.550 (29.725, 35.600)	35.700 (33.975, 41.025)	0.0042
**PT (median, IQR)**		14.350 (13.675, 15.600)	15.200 (13.775, 16.475)	13.900 (13.525, 14.650)	0.0129
**AFP (median, IQR)**		69.590 (4.742, 1200.000)	89.140 (8.832, 3280.730)	47.970 (3.147, 314.475)	0.1661
**PVTT (n, %)**	Absence	34 (60.71)	13 (46.43)	21 (75.00)	0.029
	Presence	22 (39.29)	15 (53.57)	7 (25.00)	
**T stage (n, %)**	I/II	30 (53.57)	12 (42.86)	18 (64.29)	0.1803
	III/IV	26 (46.43)	16 (57.14)	10 (35.71)	

**CHILD score**	A	41 (73.21)	21 (75.00)	20 (71.43)	1
**(n, %)**	B+C	15 (26.79)	7 (25.00)	8 (28.57)	

MATERIALS AND METHODS

Orthotopic HCC mouse model

These experiments were approved by the Institutional Animal Care and Use Committee of the Third Affiliated Hospital of SYSU. C57BL/6J mice (male, aged 6–8 weeks) were purchased from the Guangdong Medical Laboratory Animal Center (Guangzhou, China). All mice were housed and maintained under pathogen-free conditions. Preparation and transportation of Hepa1-6 cell Matrigel matrix glue (Corning, Corning, NY, USA) suspension: Hepa1-6 cells were routinely cultured and transferred 3–5 generations after recovery. Hepa1-6 cells in the logarithmic growth stage were digested and centrifuged, and then prepared into a mixture containing 1×10^6 cells, 10 μL PBS, and 10 μL Matrigel matrix gel. All devices in contact with the Matrigel matrix glue were pre-cooled in a 4 °C refrigerator, and the prepared Hepa1-6 cell Matrigel matrix glue suspension was placed on ice for transport.

Operation: 10–15 min before operation, C57BL/6J male mice were anesthetized by intraperitoneal injection with 160 μL 1% pentobarbital per 25 g weight. Then, the response of the animals to anesthesia was observed. The mice should breathe slowly and shallowly and not respond to squeezing of the back feet. The abdominal skin of the mice was shaved with a shaver, and the fine fur attached to the skin was wiped with a wet gauze with 70% (v/v) ethanol to prevent the fur from entering the peritoneum. The animals were placed in the supine position and fixed on to the lid of a 100 mm Petri dish. Cotton swabs were placed on the backs of mice to better expose the liver. The skin was sterilized with povidone iodine solution, and an incision of approximately 1 cm was made in the middle of the abdomen under the xiphoid process with ophthalmic scissors. Another 1-cm incision was made along the alba line of the lower abdomen of the xiphoid process, and the middle liver was gently

pulled onto the peritoneum with a cotton swab moistened with normal saline and placed on the outer surface of the peritoneum. A cotton swab moistened with normal saline was placed on the dorsal side of the middle lobe of the liver of mice, and 20 μL Hepa1-6 cells mixed with Matrigel matrix glue were absorbed with 0.5 mL insulin injection needle. The cells were inserted near the lower edge of the liver in the middle lobe of the liver and slowly injected into the left lobe of the liver under the capsule. After injections, needles were withdrawn along their direction, and a cotton swab was used to place pressure for 1 min to stop the bleeding. Before suture, no bleeding or fluid leakage was observed at the injection site. The cotton swab was removed and the liver returned to the enterocoelia; then, the peritoneum and skin incision were closed by intermittent 5–0 suture. Then, the skin was partially wiped with a sterile gauze to remove any blood. The animal was placed on its side on a thermal blanket until the anesthetic wore off (30–60 min). When the mice were resuscitated and could roll over on their own, they were placed in a new cage.

Splenectomy mouse model

Mice were placed in the right lateral position. A 1.0–2.0 cm skin incision was made with scissors along the left side, midway between the last rib and the hip joint. The connective tissue was loosened using the blunt end of forceps. Subsequently, a 1.0–2.0 cm incision was made through the peritoneal wall. The spleen was gently placed and placed on the exterior surface of the peritoneum. The artery attached to the spleen was tied off with a 4-0 suture by looping the suture through the mesentery. A single knot at the tip of the spleen was made, and the mesentery and connective tissue were cut to remove the spleen. The peritoneal wall was closed with one or two separate sutures, and the skin was closed with an additional two or three sutures.

$CD45^+EPC$ transfer experiment

$CD45^+EPCs$ were isolated by FACS from the tumor or spleen of tumor-bearing mice. Then $CD45^+EPCs$ were stained with CFSE (2.5 μM) for 10 min at 37 °C. Next, 1.5×10^6 $CD45^+EPCs$ from the tumor or spleen were transferred into splenectomized tumor-bearing mice via caudal vein injection. After 12 h, mice were sacrificed, and $CFSE^+$ $CD45^+EPCs$ were assessed in the blood and tumor tissue. Other mice were sacrificed after 20 days for analysis of MVI or further survival assessment.

$CD45^+EPC$ migration analysis

The bottom of a 24-well plate was coated with 160 μL complete medium + 20 μL Matrigel matrix glue, 160 μL complete medium containing 1×10^6 Hepa1-6 cells + 20 μL matrix glue, 160 μL complete medium containing 1×10^6 F4/80 positive cells + 20 μL matrix glue, or 160 μL containing 1×10^6/well of F4/80 negative cells + 20 μL matrix glue. The plate was incubated at 37 °C for 30 min to solidify the matrix glue. Then, 420 μL complete medium was mixed with the substrate glue, and 200 μL suspension complete medium containing 5.0×10^5 $CD45^+EPCs$ was added to each well of a Transwell chamber. The chamber was removed 48 h later, and the cell density of the indoor medium was counted. After counting, the medium was frozen at -80 °C.

Cell migration and invasion analysis

Migration or invasion assays were performed using 24-well plates with 8.0-μm pore size Transwell filter inserts (Corning) with or without pre-coated diluted Matrigel (Corning). Then, 2×10^4 C166 or Hepa1-6 cells with serum-free medium were placed into the upper chamber, and medium containing 20% (v/v) FBS was added to the bottom chamber. After incubation at 37 °C for 24 h (migration) or 72 h (invasion), cells on the underside of the

membrane were immobilized and stained with crystal violet (Solarbio Life Sciences, Beijing, China). Then, penetrated cells were counted in five random fields under the microscope. TGF-β-1,2,3 antibody (Invitrogen/eBioscience, Carlsbad, CA, USA) was used to block TGF-β *in vitro*.

Vascular endothelial cell tube formation analysis assay

The 96-well plates were coated with 20 μL per well of Matrigel (Corning). C166 cells were serum-depleted overnight in DMEM and then seeded at 1.5×10^4 cells per well and allowed to attach for 45 min. The medium was then replaced with conditioned medium of $CD45^+$EPCs from the spleen and tumor tissues of tumor-bearing mice. C166 cells on this matrix migrated and formed tubules within 12 h of plating. Tubule formation was monitored at 40× magnification using a light microscope (Nikon Eclipse TS100, Surbiton, UK) For the experiments using $\alpha_v\beta_3$, the conditioned medium of $CD45^+$EPC from the spleen and tumor tissues of tumor-bearing mice was incubated with recombinant $\alpha_v\beta_3$ for 1 h before addition to the Matrigel assay.

Cell proliferation assay

Cell proliferation ability was measured with a Cell Counting Kit-8 (CCK-8, Dojindo Laboratories, Kumamoto, Japan). 2×10^3 C166 or Hepa1-6 cells were seeded into a 96-well plate per well with three duplications, followed by coculture with $CD45^+$EPCs for 24, 36, 48, or 72 h. $CD45^+$EPCs were removed at the indicated times, and C166 or Hepa1-6 cells were incubated with CCK-8 for 2 h at 37 °C. Absorbance was detected at 450 nm.

Construction of stable knockdown and overexpressed cells

Lentiviruses expressing shWtap, shCebpb, shNC, AAV-Wtap, AAV-Cebpb, or empty

AAV vector were purchased from GeneChem, Co. Ltd. (Shanghai, China). Mouse tumor $CD45^+$EPCs were selected to establish stable *Wtap*-knockdown models (Table S4), while the spleen $CD45^+$EPCs of cancer mice were used for stable AAV-Wtap overexpression experiments. According to the supplier's instructions, 10^5 cells in 100 μL StemSpan SFEM II medium per well were planted into a 96-well plate and transfected with the indicated virus (final MOI = 50) for 24 h. The transfection efficiency was determined by flow cytometric analysis. The cells were then washed twice with 1× PBS and replated in StemSpan SFEM II medium at 250,000/mL.

In vitro maturation of $CD45^+$EPCs.

We cultured 1×10^5 $CD45^+$EPCs from mouse BM, liver, or spleen in 100 μL StemSpan^{TM} SFEMII medium supplemented with 10 IU/mL human recombinant erythropoietin. The cultures were maintained at 37 °C in a 5% CO_2-humidified atmosphere in 96-well plates for 6 days. Wright–Giemsa staining was performed to examine cell morphology.

Flow cytometry analysis and sorting

Cell phenotypes were evaluated on CytoFLEXLX (Beckman Coulter Life Sciences, Brea, CA, USA), and data were analyzed with the CytExpert and FlowJo V10.0.7 (FlowJo, Ashland, OR, USA). Data were acquired as the fraction of labeled cells within a live-cell gate set to a minimum of 50,000 events. For flow cytometric sorting, a BD FACSAria III cell sorter (BD Bioscience) was used. The antibodies used are listed in Table S2. The gating strategy is shown in Fig. S6–8.

Isolation of PBMCs, cord blood mononuclear cells (CBMCs), and mononuclear cells from liver, BM, and spleen.

Blood and tissue samples were analyzed within 6 h after sampling. PBMCs and CBMCs were isolated from whole blood or cord blood through Ficoll centrifugation. Liver samples were cut into approximately 1-mm^3 pieces and were triturated with a 1-mL syringe plunger on a 70-μm Cell-Strainer (BD) in RPMI-1640 medium (Invitrogen) containing 10% (v/v) FBS until uniform cell suspensions were obtained. After the supernatant was removed, the pellets were washed twice with PBS. Later, cells were resuspended with 7 mL 30% Percoll (GE Healthcare, 17089109-1) and then paved upon 3 mL 70% Percoll at the bottom of a 15-mL tube. The cells were sequentially centrifuged in an Eppendorf 5810R centrifuge at 600 *g* for 20 min. Liver mononuclear cells were collected from the cell layer at the interface between 30% and 70% Percoll. Mouse BM cells were harvested by femoral bone flushing and filtered through a 70-μm pore size cell strainer (Corning). Spleens were mechanically dissociated and filtered.

Intracellular staining

Freshly isolated mononuclear cells were incubated with Cell Stimulation Cocktail (eBioscience, Cat #00-4970-30) for 4 h at 37 °C. Then, the mononuclear cells were harvested, stained with antibodies for surface markers, and permeabilized using the Intracellular Fixation & Permeabilization BUFFER Set Kit (eBioscience, Cat#88-8824-00) overnight at 4 °C. After washing with PBS, the cells were incubated and additionally stained for intracellular cytokine antibodies. The labeled cells were analyzed via flow cytometry.

Immunofluorescence analysis

Liver tissues were successively cut in one direction from the liver right lobe for mouse experiments and used for paraffin embedding. Liver tissues of patients with HCC were obtained from those who underwent surgery. Normal liver tissues from surgery of patients

with liver hemangioma were used as the control. Paraffin-embedded sections from liver tissues were deparaffinized with xylene and rehydrated with descending grades of ethanol. Immunofluorescence was performed after antigen retrieval. Sections were microwaved in 10 mM citric acid for 2 min at high power followed by 5 min at 50% power. The sections were then cooled for 60 min at room temperature, washed in PBS, and blocked with 0.5% BSA/PBS for 30 min at 37 °C. Later, the sections were incubated with primary antibodies (listed in Table S2) overnight at 4 °C and then incubated for 30 min at 37 °C. After washing in PBS, the sections were incubated with an appropriate secondary antibody, 1:400 diluted goat/donkey anti-rat Alexa Fluor 488, goat/donkey anti-rabbit Alexa Fluor 594, or goat/donkey anti-mouse Alexa Fluor 647 (listed in Table S2) for 45 min at 37 °C. Finally, the sections were washed and mounted with DAPI (Mounting Medium with DAPI – Aqueous, Fluor shield Abcam, Cambridge, MA, USA; ab104139) and stored in dark at -30 °C until imaging. Identical confocal settings were used to limit intra-experimental variability. Fluorescent signals were detected using a laser scanning confocal microscope (ZEISS LSM 780) and automatic slide slice scan (Panoramic MIDI, 3DHISTECH). For the quantification of immunofluorescence results, the images of the indicated tissues were analyzed with Imaris 8.4 (BITPLANE). Alternatively, the absolute numbers of the CD71 and CD235a/Ter119 double positive cells/mm^2 in nine fields of interested areas were statistically analyzed. Investigators were blinded to the experimental group when performing these studies.

Measurement of intracellular ROS

ROS levels were determined through 2′,7′-dichlorofluorescein diacetate (DCFDA) staining. Briefly, mononuclear cells were incubated with 2.5 μM DCFDA at 37 °C for 30 min. Cells were then washed three times, resuspended in PBS, and stained with surface markers at 4 °C for 30 min. ROS levels were analyzed using a flow cytometer at excitation

and emission wave lengths of 490 and 520 nm, respectively.

ELISA

Culture supernatants of macrophage and target cell co-culture system were collected for ELISA. The levels of indicated cytokines (Table S3) in the culture supernatants was determined through ELISA following the manufacturer's instructions.

RNA-binding protein immunoprecipitation (RIP)

The RIP assay was performed using the Magna RIP™ RNA Binding Protein Immunoprecipitation Kit (Millipore, 17–700) according to the manufacturer's instructions. Briefly, cells were lysed in RIP lysis buffer on ice for 30 min. After centrifugation, the supernatant was incubated with 30 μL of protein-A/G agarose beads (Roche, Indianapolis, IN, USA) and antibodies at 4 °C overnight with rotation. The next day, the immune complexes were centrifuged then washed six times with washing buffer. The bead-bound proteins were further analyzed using western blotting. The immunoprecipitated RNA was applied to qRT-PCR analysis.

Nuclear and cytoplasmic extraction

Cytoplasmic and nuclear fractions were isolated as instructed by the manufacturer, using the reagents supplied in PARISKit (AM1556, Thermo Fisher Scientific). Briefly, $CD45^{+}$EPC was lysed in cell fraction buffer on ice for 10 min. After centrifugation at 500 × *g* for 5 min at 4 °C, the supernatant was collected as the cytoplasmic fraction. Then, after washing the pellet with cell fraction buffer, the nuclei were collected.

Treatment of methylation inhibitors

CD45$^+$EPCs were treated with DAA (B6121, APExBIO) with concentrations of 0 or 100 μM for 12 h, followed by the RT-qPCR or western blotting analysis to examine the expression of indicated genes.

Graphic abstract

Graphic abstract was drawn using BioRender (https://www.biorender.com).
